# Phylodynamic Inference and Model Assessment with Approximate Bayesian Computation: Influenza as a Case Study

**DOI:** 10.1371/journal.pcbi.1002835

**Published:** 2012-12-27

**Authors:** Oliver Ratmann, Gé Donker, Adam Meijer, Christophe Fraser, Katia Koelle

**Affiliations:** 1 Department of Biology, Duke University, Durham, North Carolina, United States of America; 2 Department of Infectious Disease Epidemiology, Imperial College London, London, United Kingdom; 3 NIVEL, Netherlands Institute for Health Services Research, Utrecht, The Netherlands; 4 RIVM, National Institute for Public Health and the Environment, Centre for Infectious Disease Control, Bilthoven, The Netherlands; 5 Fogarty International Center, National Institutes of Health, Bethesda, Maryland, United States of America; University of California San Diego, United States of America

## Abstract

A key priority in infectious disease research is to understand the ecological and evolutionary drivers of viral diseases from data on disease incidence as well as viral genetic and antigenic variation. We propose using a simulation-based, Bayesian method known as Approximate Bayesian Computation (ABC) to fit and assess phylodynamic models that simulate pathogen evolution and ecology against summaries of these data. We illustrate the versatility of the method by analyzing two spatial models describing the phylodynamics of interpandemic human influenza virus subtype A(H3N2). The first model captures antigenic drift phenomenologically with continuously waning immunity, and the second epochal evolution model describes the replacement of major, relatively long-lived antigenic clusters. Combining features of long-term surveillance data from the Netherlands with features of influenza A (H3N2) hemagglutinin gene sequences sampled in northern Europe, key phylodynamic parameters can be estimated with ABC. Goodness-of-fit analyses reveal that the irregularity in interannual incidence and H3N2's ladder-like hemagglutinin phylogeny are quantitatively only reproduced under the epochal evolution model within a spatial context. However, the concomitant incidence dynamics result in a very large reproductive number and are not consistent with empirical estimates of H3N2's population level attack rate. These results demonstrate that the interactions between the evolutionary and ecological processes impose multiple quantitative constraints on the phylodynamic trajectories of influenza A(H3N2), so that sequence and surveillance data can be used synergistically. ABC, one of several data synthesis approaches, can easily interface a broad class of phylodynamic models with various types of data but requires careful calibration of the summaries and tolerance parameters.

## Author Summary

The infectious disease dynamics of many viral pathogens like influenza, norovirus and coronavirus are inextricably tied to their evolution. This interaction between evolutionary and ecological processes complicates our ability to understand the infectious disease behavior of rapidly evolving pathogens. Most statistical methods for the analysis of these “phylodynamics” require that the likelihood of the data can be explicitly calculated. Currently, this is not possible for many phylodynamic models, so that questions on the interaction between viral variants cannot be well-addressed within this framework. Simulation-based statistical methods circumvent likelihood calculations. Considering interpandemic human influenza A virus subtype H3N2, we here illustrate the effectiveness of these methods to fit and assess complex phylodynamic models against both sequence and surveillance data. We find that combining molecular genetic and epidemiological data is key to estimate phylodynamic parameters reliably. Moreover, the information in the available data taken together is enough to expose quantitative model inconsistencies. Methods such as ABC which can combine sequence and surveillance data appear to be well-suited to fit and assess mechanistic hypotheses on the phylodynamics of RNA viruses.

## Introduction

Many infectious pathogens, most notably RNA viruses, evolve on the same time scale as their ecological dynamics [1]. One of the perhaps best documented examples are human influenza A viruses, which cause substantial morbidity and mortality as they escape host immunity predominantly through the evolution of their surface antigens [2]. The resulting, dynamical interaction between the ecological and evolutionary processess can be better understood through the formulation and simulation of so-called “phylodynamic” mathematical models, e.g. [3]–[8]. However, while data on disease incidence as well as viral genetic and antigenic variation are increasing for many viruses, e.g. [9]–[13], fitting and assessing phylodynamic models to these data is still not commonly done.

Historically, epidemiological time series data have been pervasively used to analyze hypotheses of host-pathogen interactions at the population level [14]–[17]. However, time series data capture the underlying evolutionary processes of pathogens only very indirectly. For flu, this has limited the type of infectious disease models that can be statistically interfaced with time series data, and the number of epidemiological parameters that can be simultaneously estimated [18], [19]. Consequently, the disease behavior of rapidly evolving pathogens is increasingly studied under additional, complementary data sets [1], most typically in ways that attempt to qualitatively reproduce prominent disease attributes [3]–[8].

More recently, coalescent-based statistical methods have been used to elucidate the disease dynamics of RNA viruses from molecular genetic data alone [20]. These methods have been particularly useful to reconstruct epidemiological transmission histories, identifying when and where transmission occurred and how viral populations change over time. For example, coalescent-based analyses have highlighted the importance of the tropics in the complex circulation dynamics of human influenza A (H3N2) virus (in short: H3N2) [9], [21], [22]. However, most coalescent methods estimate past population dynamics within a class of flexible demographic functions including exponential and logistic growth as well as the nonparametric Bayesian skyride [23], [24]; but see also [25]. These demographic functions do not explicitly describe the non-linear population dynamics of RNA viruses. Thus, assessing which ecological interactions underlie observed patterns of sequence diversity, and estimating the respective strength of these interactions, is difficult within this framework.

Because of these limitations, we adopt a different statistical approach known as Approximate Bayesian Computation (ABC) to infer the phylodynamics of RNA viruses. ABC allows mechanistic phylodynamic models to be simultaneously fitted against both sequence and surveillance data. This method circumvents explicit likelihood calculations by simulating instead from the stochastic model that defines the likelihood [26]. Recent extensions of ABC allow for model assessment to be carried out at no further computational cost [27]. We further suggest incorporating variable selection procedures to quantify if and to what extent the data provide support for the inclusion of specific model components [28].

**Figure 1:**
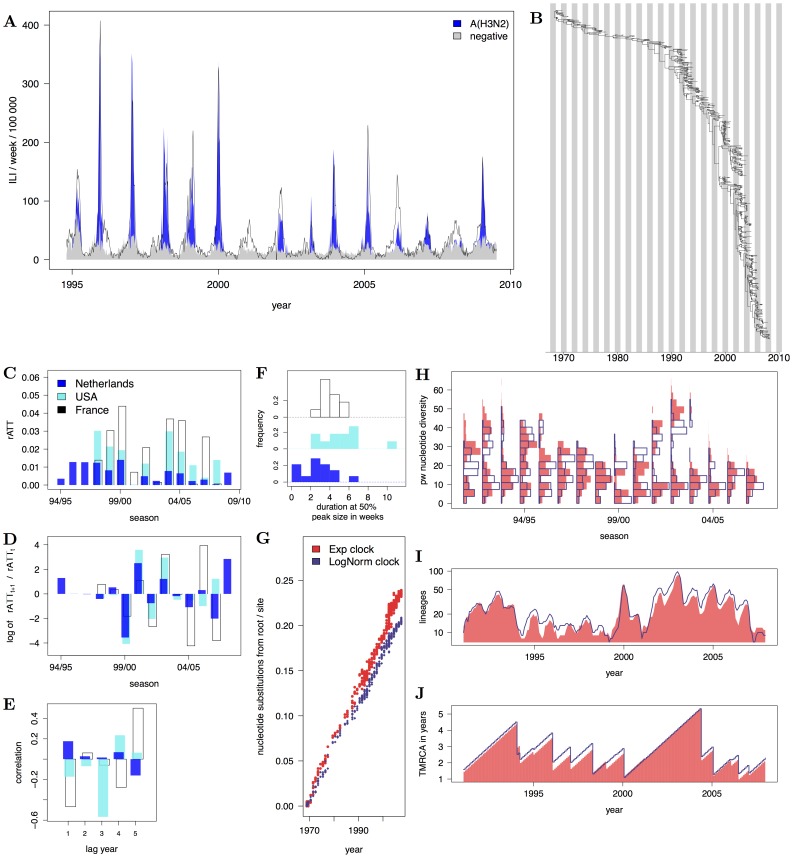
Features of H3N2 sequence and incidence data. (A) Weekly ILI time series from the Netherlands, and estimated time series of influenza A(H3N2) from weekly virological data. Type and subtype specific time series were estimated under an additive Negative Binomial regression model; see Text S1. (B) Reconstructed HA phylogeny from 776 European sequences with known times of isolation. The phylogeny was inferred with the BEAST program under a relaxed Exponential clock; see Text S1. (C) H3N2 seasonal attack rates (rATT), calculated from estimated H3N2 case report times series in the Netherlands in 1994–2009 (blue), and the USA (cyan) as well as France in 1997–2008 (black). (D) Ratio of consecutive case report attack rates on the log scale. (E) Autocorrelation of case report peaks. (F) Histogram of the duration of seasonal epidemics at half their peak size. (G) Number of estimated nucleotide substitutions of dated HA sequences from the root A/Bilthoven/16190/68 as in Smith et al. [31]. Nucleotide substitutions were estimated with BEAST under an Exponential clock (red) and Lognormal clock model (violet). (H) Histograms of pairwise nucleotide diversity among sequences collected in the same season. (I) Time series of the number of phylogenetic lineages circulating within the same month. (J) Time series of the time to the most recent common ancestor of phylogenetic lineages circulating within the same month. Colors from H to J are as in G.

To demonstrate the utility of our approach, we consider the phylodynamics of interpandemic H3N2. We obtained weekly reports of H3N2 incidence in the Netherlands from 1994–2009 by combining influenza-like-illness (ILI) surveillance data with detailed records of associated, laboratory-confirmed cases of flu by type and subtype [29], [30], and similarly for France and the USA; see Figure 1 and the supplementary online material (Text S1). In addition, we reconstructed the ladder-like phylogeny of H3N2's haemagglutinin gene (HA) from dated European sequences collected in 1968–2009 (see Figure 1 and Text S1). To represent H3N2's global phylodynamics, we focus on a class of spatially structured phylodynamic compartmental models that formalize probabilistically how evolving, antigenic variants interact epidemiologically. These antigenic variants might correspond to the major antigenic clusters that are distinguishable in H3N2 antigenic maps [31], but can in principle also represent a different phenotypic resolution. The evolutionary dynamics of viral genotypes are separately formulated for each antigenic phenotype because genetic distances do not necessarily easily translate into phenotypic relationships [5]. Spatial substructure has been incorporated in several models of H3N2 phylodynamics to reflect the global circulation of the virus [4], [8], [32]. We adopt here a simple source-sink framework, where the sink is thought of as the Netherlands into which viral genetic diversity and antigenic strains are imported on a seasonal scale from a source population where the virus persists [9], [33]. We fit and assess two distinct models to the combined features of sequence and incidence data described in Figure 1 and Table 1. The first model captures H3N2's antigenic drift phenomenologically through gradual loss of immunity, and the second model describes the antigenic evolution of the virus explicitly with particular assumptions on the tempo of antigenic change.

**Table 1:** Basic phylodynamic summaries of H3N2 surveillance data and phylogenies, and calibrated weighting schemes.

shorthand	summary	data	distance	summary values and distances*			weighting scheme†	
				Netherlands	France	USA	under the SEIRS model	under the epochal evolution model
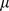 *-attack*	average 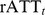 , where 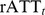 is the total case report incidence in season 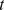		log ratio	0.56%	1.9% (−1.26)	1.4%(−0.97)	Indicator (3)	Indicator (3)
							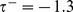	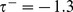
							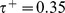	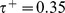
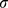 *-attack*	standard deviation in 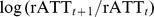		log ratio	1.68	2.78 (−0.5)	2.24 (−0.28)	Indicator (3)	Indicator (3)
							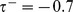	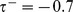
							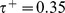	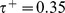
*explosiveness*	average duration of reported seasonal epidemics at half their peak size	time series 1994–2009	log ratio	3.2	4.54 (−0.32)	5.81 (−0.57)	Indicator (3)	Indicator (3)
							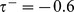	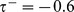
							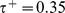	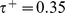
*correlation*	Pearson autocorrelation of case report peaks at a lag of 2 & 4 years		largest difference	0.07 & 0	0.06 & −0.27 (−0.27)	−0.06 & 0.23 (0.23)	Exponential (4)	Indicator (3)
							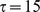	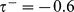
								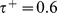
*pop-attack*	largest seasonal population-level attack rate	Ref. [2]	difference 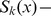 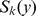		20%		Indicator (3)	Exponential (4)
							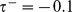	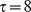
							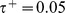	

* Distances between summaries derived from the first listed and subsequent data sets are given in brackets.

† Weighting schemes differ across models to accommodate weak or strong inconsistencies; see also Table 3.

‡ The number of dated HA sequences available before 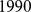 is very small, so that these years effectively do not contribute to the *diversity*. To make this sampling effect more apparent, all phylogenetic summaries except the *divergence* are only computed on the period 1991–2009.

## Methods

### Approximate Bayesian Computation

To perform phylodynamic inference and goodness-of-fit analyses for complex phylodynamic models, we adopt a simulation-based approach that has become known as Approximate Bayesian Computation (ABC) [26]. Our first goal is to estimate the posterior density
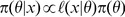
(1)of epidemiological and evolutionary model parameters 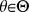 under approximations to the likelihood 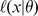 of observed population incidence and phylogenetic data 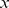. The prior density 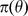 can be used to incorporate existing information or limit the range of plausible values of model parameters. Our second goal is to assess fitted phylodynamic models based on a recent extension of ABC [27].

**Figure 2:**
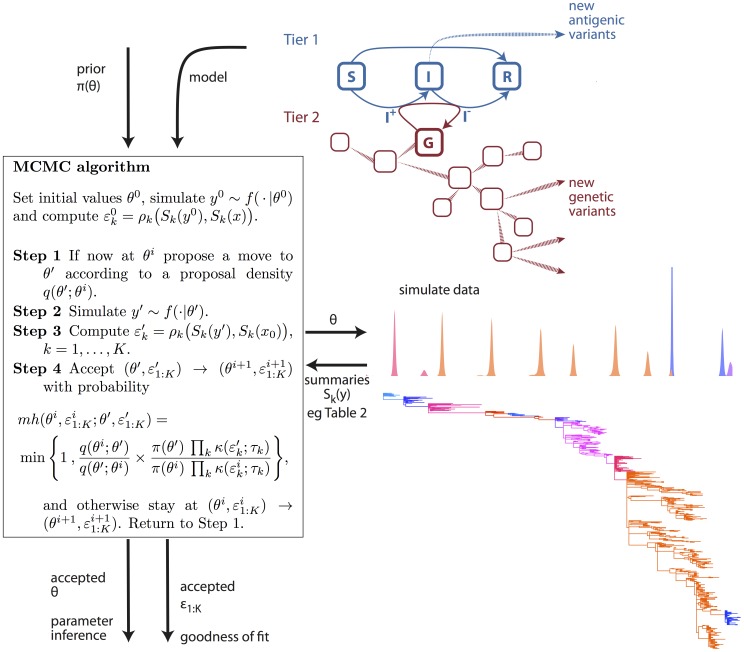
Overview of simulation-based phylodynamic inference and model assessment. Phylodynamic hypotheses are formulated into evolving, dynamical systems models. We used a two-tier model formulation whose genetic component is tied to its ecological component through the flows through the prevalence class. Existing knowledge on model parameters is incorporated through the prior 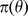, and Monte Carlo algorithms such as MCMC are used to fit the model to different types of data, e.g. incidence time series and reconstructed phylogenies (see Figure 1) with an ABC approach. ABC is based on likelihood approximations such as (2), which requires a specification of phylodynamic summaries (e.g. Table 1). The summary errors are used to diagnose if the fitted phylodynamic model is consistent with available data in terms of the specified summaries.

ABC methods circumvent computations of the likelihood 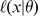 by comparing the observed data 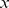 to simulated data 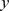 in terms of many, lower-dimensional summary statistics 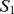, 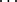, 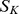 such as those in Figure 1. Using a distance function 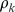 that compares summaries, each simulation 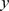 is weighted according to the magnitude of the summary error 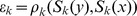 under a weighting scheme 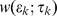, and this value is used in place of the likelihood term in Monte Carlo algorithms. In essence, ABC is a particular auxiliary variable Monte Carlo method, where the 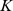 summary errors take on the role of auxiliary variables. Integrating these errors out, the ABC likelihood approximation 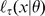 adopted here is
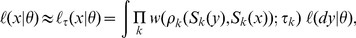
(2)where the weighting scheme is typically the Indicator
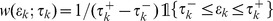
(3)with tolerance parameter 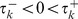 or the Exponential
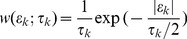
(4)with 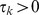. Intuitively, the summary errors indicate how well a parameterized model reproduces the observed data. Once Monte Carlo algorithms such as the Markov Chain Monte Carlo (MCMC) sampler proposed by Marjoram et al. [34] have converged, the magnitude of the summary errors can be used to diagnose goodness-of-fit with respect to each of the summaries 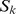. To use this detailed information on each summary, we prefer using (2) to the Mahanalobis approximation (see [26]). Although uncommon, we typically use the log ratio 
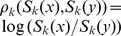
 so that the errors 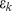 can be uniformly interpreted as fold-deviations. Parameter inference using ABC is approximate in that the ABC target density 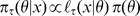 approaches the posterior density (1) as 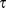 tends to zero if the summaries are sufficient for 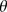 [26]. We use a Monte Carlo algorithm that is very similar to the MCMC sampler in Figure 2. A full specification of the algorithm is given in Text S1.

It is typically difficult to establish the sufficiency of phylodynamic summaries analytically, and instead a small set of summaries is chosen such that model parameters of interest can be estimated [26]. Table 1 lists basic features of H3N2 epidemiological and phylogenetic data that were primarily considered in this study. Phylodynamic models were fitted and assessed against the features of the Dutch incidence data and the viral phylogeny derived under the Exponential clock model. The differences between these summaries and those derived from the remaining data in Figure 1 were used to set the ABC tolerances large enough so that inference is robust to the choice of phylogenetic reconstruction method and reporting country. Although smaller tolerances can be computationally feasible, these were not supported by the additional data considered. We typically use the Indicator weighting scheme (3) with tolerances 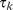 that encompass differences in summary values across reporting countries and/or reconstruction methods, see Table 1. When a model never fits a particular summary well, we use (4) to give a mild prior preference to small errors [27]. See Text S1 for further details.

### Spatial two-tier models to represent H3N2 phylodynamics

#### Deterministic skeleton

ABC methods require that each phylodynamic simulation must run on the order of tens of seconds. To meet this computational requirement while still allowing for flexible modeling [6], [35], we adopt a two-tier approach that separates the genotypes of rapidly evolving viruses from their antigenic phenotypes [7]. The underlying rationale is that differences in genotype are only relevant from a population dynamic perspective if they translate into perceivable phenotypic differences. The first tier describes the dynamic interactions of antigenic variants in the host population, here in terms of coupled susceptible-exposed-infected-recovered-susceptible (SEIRS) equations that are further spatially structured into a strongly seasonally forced sink population and a re-seeding, weakly seasonally forced source population (denoted by 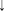 and  respectively). The second tier simulates a phylogeny that is consistent with the prevalence and incidence dynamics of each antigenic unit in the first tier. Assuming polarized immunity [3], the deterministic skeleton for the 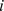
th antigenic unit is
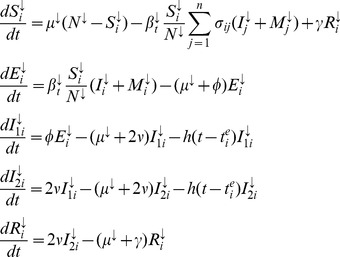
(5a)

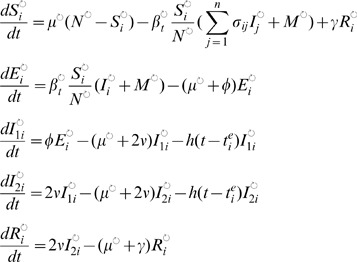
(5b)

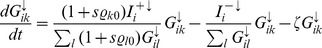
(5c)

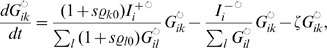
(5d)where all model parameters are described in Table 2 or below. Two infectious subcompartments 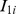, 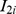 are employed to obtain more realistic infectiousness profiles [36]. 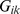 is the number of individuals infected with the 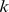
th genotype of the 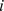
th antigenic unit, 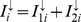, 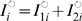 for convenience, and 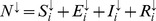 and 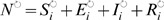 for all 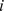.

**Table 2:** Phylodynamic model parameters, prior and estimated densities.

symbol	description	prior density	mean 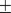 std. dev., 95% conf. interval of	
			posterior density under the SEIRS model	posterior density under the epochal evolution model
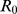	Basic reproductive number	uninformative	3.03 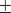 0.55, [1.77, 4.14]	18.7 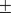 5.3, [9.2, 26.8]
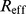	effective reproductive number	-	1.26 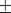 0.05, [1.17, 1.35]	1.42 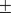 0.12, [1.27, 1.51]
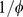	Average incubation period in days	0.9		
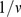	Average infectiousness period in days	1.8		
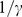	Average duration of immunity in years	uninformative	9.8 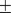 1.8, [6.5, 12.2]	206 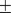 103, [46, 380]
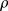	Reporting rate	uninformative	0.15 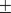 0.06, [0.06, 0.26]	0.56 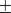 0.23, [0.25, 0.95]
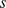	Residual selection	Exponential slab with mean 0.007 & Gaussian pseudo-prior centered at 0.09 [28]	0.1 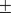 0.16, [0.01, 0.44]	0.04 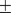 0.07, [0.001, 0.12]
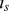	Inclusion probability of 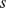	uninformative	1 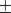 0, [1,1]	1 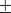 0, [1,1]
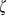	Mutation rate, 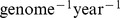	uninformative	1.32 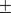 0.3, [1.0, 1.9]	3.38 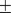 1.2, [1.8, 5.4]
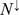	Size of sink population	fixed to Dutch demographic data, http://statline.cbs.nl		
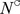	Size of source population	uninformative	1.28 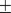 0.95, [0.43, 3.6] 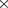 10 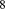	2.9 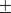 1.6, [0.7, 5.7] 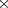 10 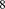
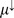	Birth/death rate in the sink population	fixed to Dutch demographic data		
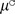	Birth/death rate in the source population, 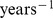	1/50; average lifespan of 60 years adjusted by net fertility rate in South East Asia		
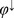	Seasonal forcing in the sink population	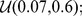 see Text S1	0.42 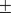 0.14, [0.3, 0.6]	0.35 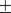 0.15, [0.12, 0.58]
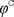	Seasonal forcing in the source population	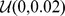 ; key assumption, see Text S1	0.01 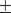 0.007, [0.002, 0.02]	0.013 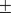 0.006, [0.008,0.02]
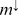	Number of travelers visiting the sink population	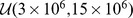 ; encompassing lowest & highest annual records; http://statline.cbs.nl	8.5 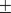 2.8, [3.6, 14.1] 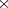 10 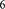	9.9 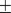 3.4, [3.8, 14.6] 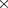 10 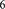
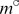	Fraction of 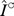 re-seeding the source population	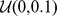	0.06 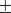 0.03, [0.01, 0.09]	0.06 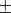 0.03, [0.02, 0.09]
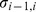	Partial cross-immunity of mother-daughter variants	uninformative	-	0.76 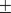 0.05, [0.67, 0.85]
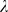	Scale parameter of the antigenic emergence rate	uninformative	-	386 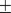 97, [247, 533]
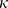	Shape parameter of the antigenic emergence rate	2; Ref. [7]		

In the first tier (5a–5b), competition between two antigenic variants 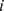, 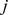 arises through resource depletion via partial cross-immunity 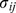 that decays multiplicatively with kinship level 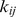, 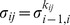, where 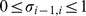 is the degree of cross-immunity between mother-daughter variants [3]. The emergence of antigenic variants is described phenomenologically with a per capita hazard function 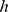 after the emergence time 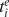 of the resident phenotype 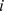 [7]. The hazard function is parameterized with a scale parameter 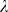 and a shape parameter 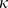. The strength of sinusoidal seasonal forcing in the source population in the transmission parameter, 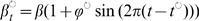, is assumed to be much smaller than in the asynchroneously forced sink population, 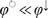, and 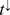 is set so that transmission peaks at the winter solstice in the Northern hemisphere. 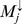 is the number of infected visiting travelers from the source, while 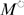 is the number of individuals that re-seed the source population. Thus, the source population can be interpreted as an interconnected, re-seeding tropical region whose population size 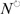 is to be estimated. We further calibrate the sink population to represent the Netherlands, using demographic data to specify 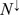 and 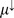 over the study period 1968–2009. To fit model (5), we transform 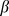 into 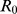 at disease equilibrium of a single variant [14], and define 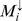, 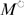 by 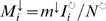 and 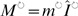 where 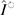 is the number of infected individuals at disease equilibrium of a single variant.

In the second tier (5c–5d), the instantaneous loss in 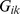 is proportional to genotype frequency, while the gain in 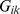 is weighted by the fitness advantage of each genotype. As before [7], fitness is assumed to increase linearly with the number 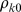 of nucleotide mutations between the 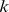
th genotype and the founder genotype of the 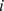
th antigenic variant. The total number of infections and losses 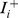 and 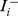 are the simulated transitions in and out of 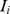 at time 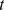, so that (5c–5d) are tied to (5a–5b). New genotypes evolve at a rate 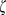, and a genealogy of the 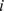
th antigenic unit is generated by recording the emergence times of each genotype along with their kinships. The branch length between offspring and parental genotype is always one. After extinct genotypes are pruned, the branch length between two genotypes gives the number of nucleotide substitutions between them. These genealogies are concatenated by connecting the root genotype with a genotype of the parental antigenic unit that is randomly drawn according to genotype frequencies at time 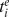. The residual selection parameter 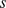 accounts phenomenologically for selection pressures between genetic variants that are evident from the shape of the virus phylogeny, but remain unexplained by a particular ecological model of antigenic variants (5a–5b). Once the distribution of 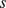 has been inferred from population incidence and genetic data, we can then quantify how well a phylodynamic model describes patterns of continual immune selection mechanistically, and also compare alternative phylodynamic models in this respect.

#### Stochastic process model

To account for demographic stochasticity, Markov transition probabilites are derived from (5), assuming that the per capita rates are constant over a small time interval 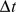, and that transitions out of any state are independent and multinomially distributed. Generally, consider a state 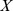 and all 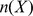 per capita rates 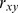 out of 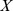. The state transitions out of 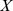 into states 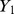,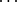,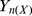 are
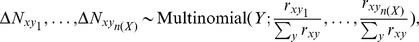
where 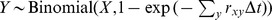 is the total number of individuals leaving 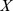 at time 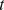.

For the application to H3N2 phylodynamics, simulations were started in 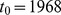 at the disease equilibrium of a single antigenic variant and generated under a multinomial Euler scheme with 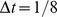 days. After the simulations of the first tier completed, the corresponding phylogeny was simulated based on the flows in and out of the prevalence compartments [7]. Simulated data were recorded after 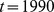 to match the time range of the observed summaries in Figure 1. We do not estimate the initial conditions of the state variables and assume that by 1990, the phylodynamic processes do not depend any longer on the initial values in 1968.

#### Stochastic observation model

To interface the two-tier model with observed case report data and phylogenies, we simulated reported incidence under a Poisson model with mean 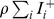 and drew a requested number of genotypes at specified sampling times without replacement according to the genotype frequencies at those times. Replacing the genotype emergence times with the corresponding sampling times and pruning non-sampled genotypes, we obtained a dated phylogeny with branch lengths encoding nucleotide substitution distances.

### Inferring inclusion probabilities of model parameters

A frequent problem in phylodynamic modeling is to determine if a specific model parameter should be included. For example, it can be unclear which types of ecological interactions between antigenic variants underlie pathogen phylodynamics, or if the residual selection parameter 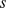 in (5c–5d) is required in addition to a given ecological mechanism that induces immune selection. Following existing variable selection procedures [28], we use an additional indicator variable 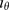 to denote whether a single model parameter 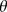 is present (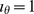) or absent (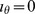) and estimate its posterior probability under equation (2). Here, we use a standard spike-and-slab variable selection procedure [28] to estimate inclusion probabilities of the residual selection parameter 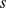.

## Results

### Basic geographic framework for modeling H3N2 phylodynamics

To illustrate ABC methodology with the summaries in Table 1, we begin with a classical phenomenological model that implicitly accounts for antigenic drift through gradual loss of immunity [37]. H3N2 phylodynamics are represented with a spatial two-tier system of equations that is a special case of (5) when the antigenic emergence rate is set to
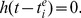
(6)For simplicity, we will refer to (5) without antigenic variants as the SEIRS model.

#### Simulated data

We first tested ABC on simulated data generated under the SEIRS model and found that the subset 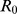, 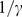, 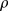, 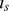, 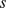, 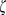 of model parameters can be reliably estimated with ABC tolerances 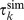 that are smaller than those in Table 1 (see Text S1). Tigher tolerances on the population level attack rate contributed most to more reliable estimates of 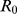.

#### Parameter inference

**Figure 3:**
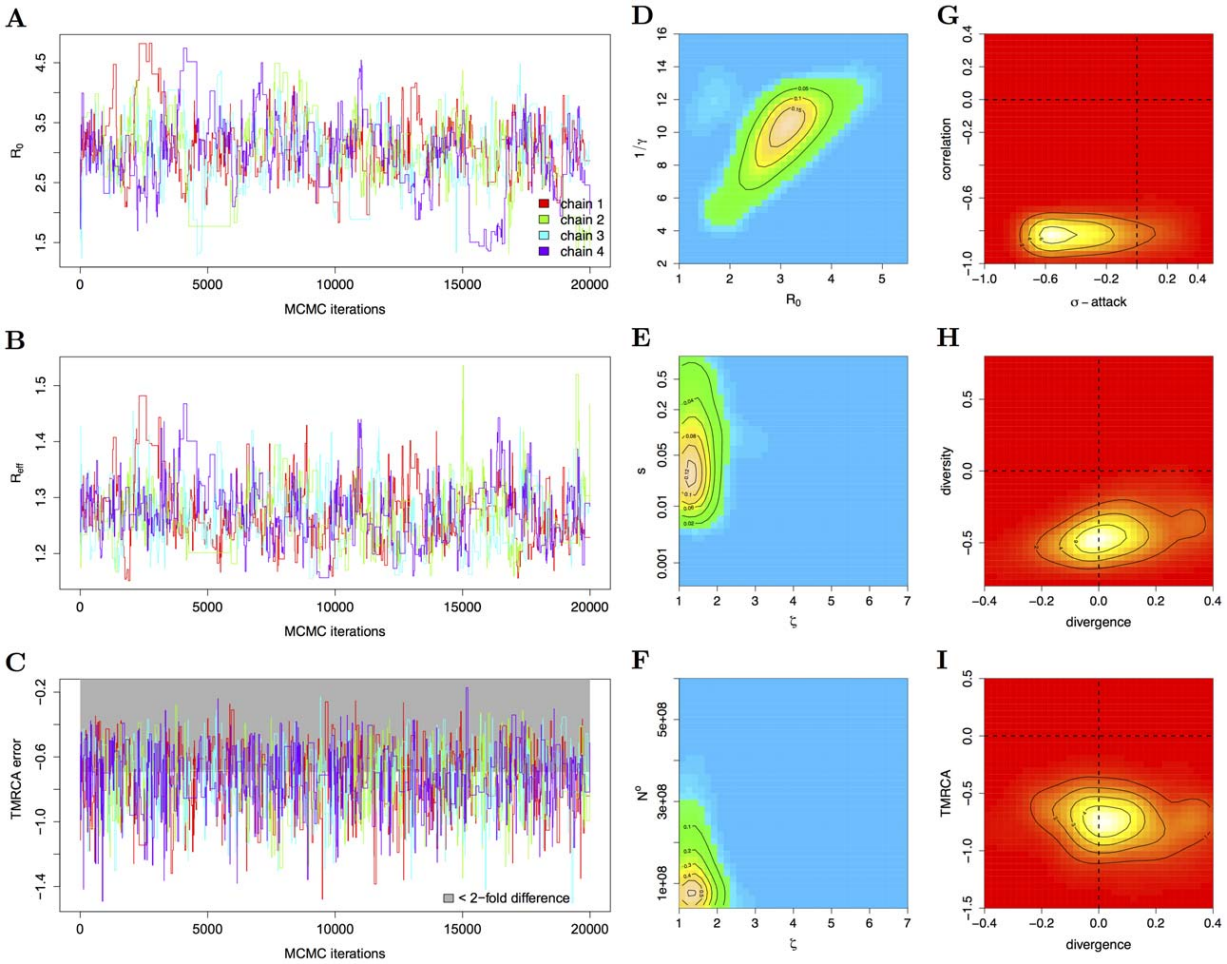
Phylodynamic inference and goodness-of-fit analysis of the spatial SEIRS model. (A–C) MCMC trajectories of the estimated 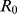, the calculated 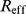, and the TMRCA summary error of four chains that were started at overdispersed starting values (see Methods). Samples before iteration 1000 were discarded. (D–F) Two-dimensional histograms of parts of the ABC fit, illustrating the correlations between the estimated parameter pairs (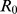, 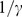), (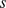, 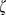) and (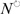,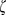). Throughout, histograms were computed from all samples across the four chains after burn-in. Color codings are separate for each subplot, with respective density values indicated in the contours. (G–I) Two-dimensional histograms of parts of the joint density of summary errors, illustrating goodness-of-fit with respect to the *correlation* and interannual variability of the case report data, as well as the *divergence*, *diversity* and the *TMRCA*'s of the HA phylogeny.

**Figure 4:**
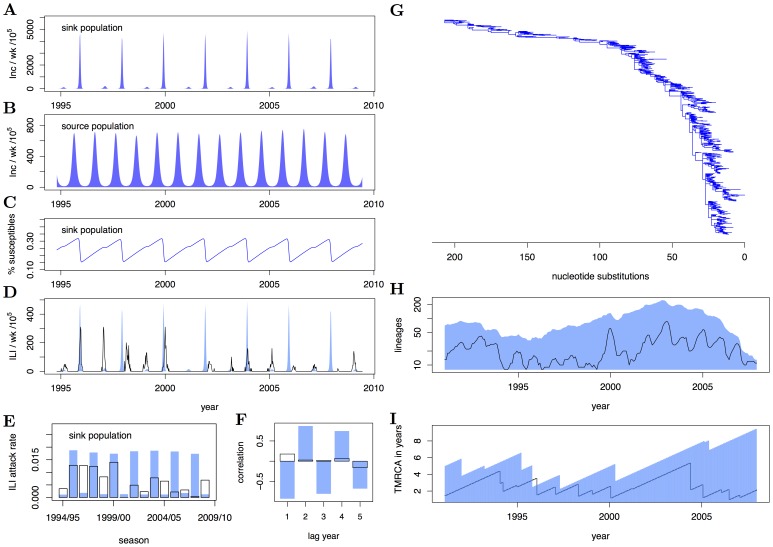
Phylodynamics arising under the spatial SEIRS model. (A–B) Population-level weakly incidence in the sink and source population, respectively. (C) Corresponding weekly time series of the percentage of susceptible individuals in the sink population. (D) Simulated H3N2 weekly surveillance time series in the sink population (blue) and reconstructed H3N2 time series in the Netherlands (black). (E) Simulated and observed case report seasonal attack rates, and (F) autocorrelation function of case report peaks. Typically, simulations under the fitted model show sustained oscillations that follow a clear biennial pattern. (G) Simulated HA phylogeny under a large, estimated residual selection parameter. (H) Simulated and observed lineage profile, and (I) simulated and observed time series of the time to the most recent common ancestor of extant phylogenetic lineages. Despite a relatively high selection parameter, the number of lineages and the time to the most recent common ancestor are overall too high when compared to data. Model parameters are 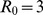, 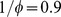, 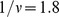, 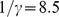, 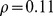, 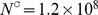, 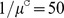, 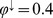, 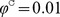, 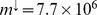, 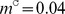, 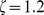, 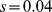.

The behavior of the spatial SEIRS model, when fitted to the case report and phylogenetic summaries in Table 1, is illustrated in Figure 3 with parameter estimates given in Table 2. On real data, the summary errors were considerably larger than on simulated data, so that the 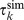 could not be used. Instead, we chose ABC tolerances with a data-driven approach that compares summary errors across different empirical data sets (see the Methods section and Table 1). Overall, we can simultaneously infer the epidemiological and evolutionary parameters 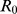, 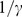, 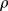, 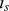, 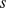, 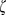, 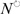. As shown in Figure 3A, the MCMC algorithm may get occasionally stuck in the tails of the target density (see Text S1 for further discussion). The posterior mean and standard deviation of 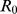, 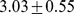, are relatively large in comparison to estimates from previous studies [36], [38]–[40], and 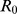 is positively correlated with the average duration of infection 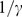 to yield realistic incidence time series. We back-calculated the effective reproductive number 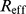 from the prevalence growth rate at the beginning of each season (see Text S1), and find that many combinations of 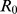 and 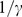 give a tight mean posterior 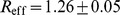 in agreement with these studies. In the absence of any ecological mechanisms inducing strain competition, the mean residual selection parameter is large 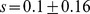 and always included in the SEIRS model. Generally, the sequence divergence imposes negative correlations between 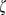, 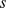 (Figure 3E) and 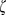, 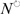 (Figure 3F), and the posterior mean mutation rate 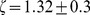/genome/year is much smaller than H3N2's substitution rate, 5.3–6.1/genome/year, when selection is incorporated into the model. Figure 4 illustrates that the fraction of susceptible individuals ranges within 15–40% and changes smoothly under seasonal forcing, thus leading to sustained oscillations in disease incidence. We failed to estimate 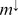, 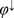, 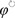, 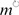 and recovered distributions close to the prior. Our prior assumptions are summarised in Table 2 and more fully discussed in Text S1.

#### Sensitivity of parameter estimates to phylodynamic summaries

The extent to which phylodynamic parameters can be estimated depends mainly on the type of information that underlies the ABC summaries. As described more fully in Text S1, a broad range of epidemiological parameters are quantitatively consistent with summaries of the H3N2 case report data in Figure 1A because variable reporting rates can mask the true extent of population incidence when immunity is not permanent [19]. Detailed studies of closely monitored populations and serological data suggest interpandemic seasonal H3N2 attack rates between 10–20% in temperate regions [2], and we found that conditioning on a broad window of maximum seasonal population incidence attack rates (*pop-attack*) between 15–30% ensures that key epidemiological parameters can be well estimated (Figure S4 in Text S1).

Moreover, while the sequence divergence and diversity are standard descriptors of viral phylogenies [1], we found that they are not sufficient to infer the size of the source population 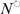 when the mutation rate 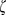 and the residual selection parameter 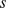 are simultaneously estimated. Considering the narrowness of the phylogeny in terms of the number of circulating lineages, we could estimate the source population size (Figures S5–6 in Text S1). We can use the number of lineages despite their dependence on sampling effort because with ABC, we are free to sample simulated sequences exactly as in the observed data set, see the Methods section. Finally, the time to the most recent common ancestor (TMRCA) links the evolutionary dynamics with the ecological interactions between antigenically distinct viral variants because weak selective advantages invariably lead to coexistence and deep phylogenies in the face of high 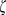 and weak 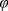. In the absence of sufficiently strong ecological interactions, the TMRCA's favor a larger residual selection parameter (Figure S7 in Text S1).

#### Goodness of fit

The summary errors reveal that the SEIRS model fails to reproduce the irregular interannual variability in winter season epidemics, and the narrowness and limited diversity of the HA phylogeny despite large 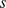 (Figure 3G–I). However, the model can reproduce H3N2's high divergence rate. This is not the case for the SEIRS model without a separate, weakly seasonally forced source population (see Text S1).

### Epochal evolution model of H3N2 phylodynamics

While several models have been able to simulate phylodynamics that are consistent with some aspects of the observed data, most notably the ladder-like phylogeny of H3N2's haemagglutinin gene [4], [5], [41], none have been quantitatively fitted and tested against a set of epidemiological and molecular genetic features such as those in Figure 1. Here, we focus on the epochal evolution model as formulated in [7] within the above spatial framework, which is identical to (5) when antigenic variants are interpreted as major antigenic clusters. To fit (5) to the serial replacement of 11 major antigenic clusters within 1968–2002 [31], we define an antigenic cluster as any antigenic unit that survives for at least 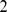 years and use the summaries in Table 1 as well as the number of antigenic clusters generated in 1968–2002 (*nclust*). Following [7], the emergence rate is set to increase with age,
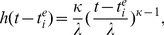
(7)

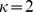
, and the scaling parameter 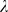 is estimated. For simplicity, we refer to (5) with this antigenic emergence rate and an antigenic resolution that is determined by *nclust* as the epochal evolution model.

#### Simulated data

We generated data under the epochal evolution model and fitted both models with the summaries in Table 1. The summary errors deviated from zero only when the SEIRS model was fitted, indicating that ABC can correctly and readily identify model mismatch (see Text S1).

#### Parameter inference

**Figure 5:**
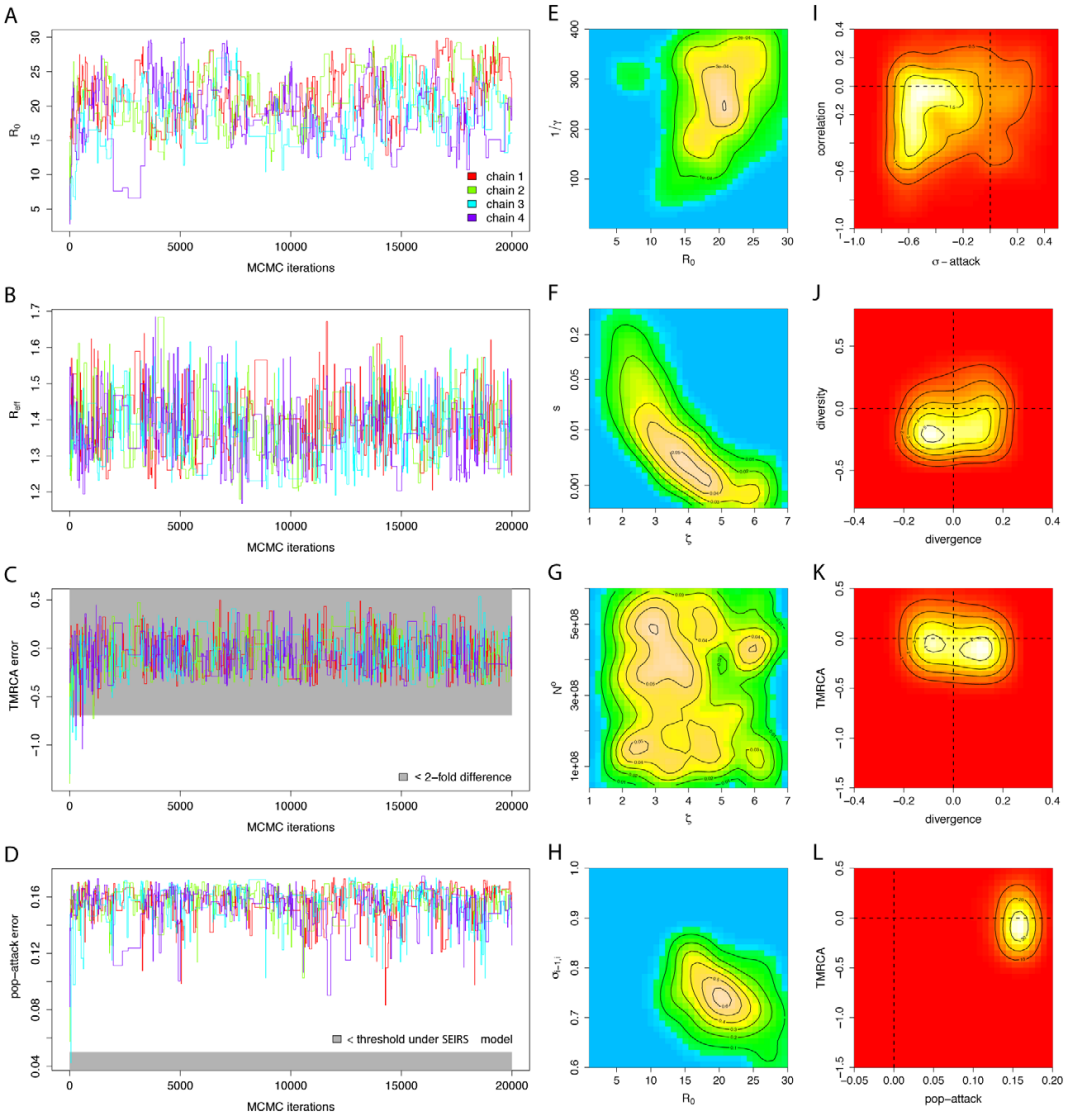
Phylodynamic inference and goodness-of-fit analysis of the spatial epochal evolution model. (A–D) MCMC trajectories as in Figure 3. The summaries *TMRCA* and *pop-attack* are in conflict and cannot be simultaneously fitted, so that the tolerance for *pop-attack* was relaxed. Samples before iteration 5000 were discarded. (E–H) Two-dimensional histograms of parts of the ABC fit as in Figure 3. Partial cross-immunity is relatively low and correlates negatively with 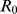. (I–L) Two-dimensional histograms of parts of the joint density of summary errors as in Figure 3. The epochal evolution model captures the irregularity in H3N2 case report attack rates, and the divergence, diversity and narrowness of the HA phylogeny well, albeit at a high residual selection parameter that is essentially always included. However, under this fitted model, population-level attack rates are in conflict with the *TMRCA*'s and cannot be simultaneously reproduced when 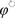 is weak.

**Figure 6:**
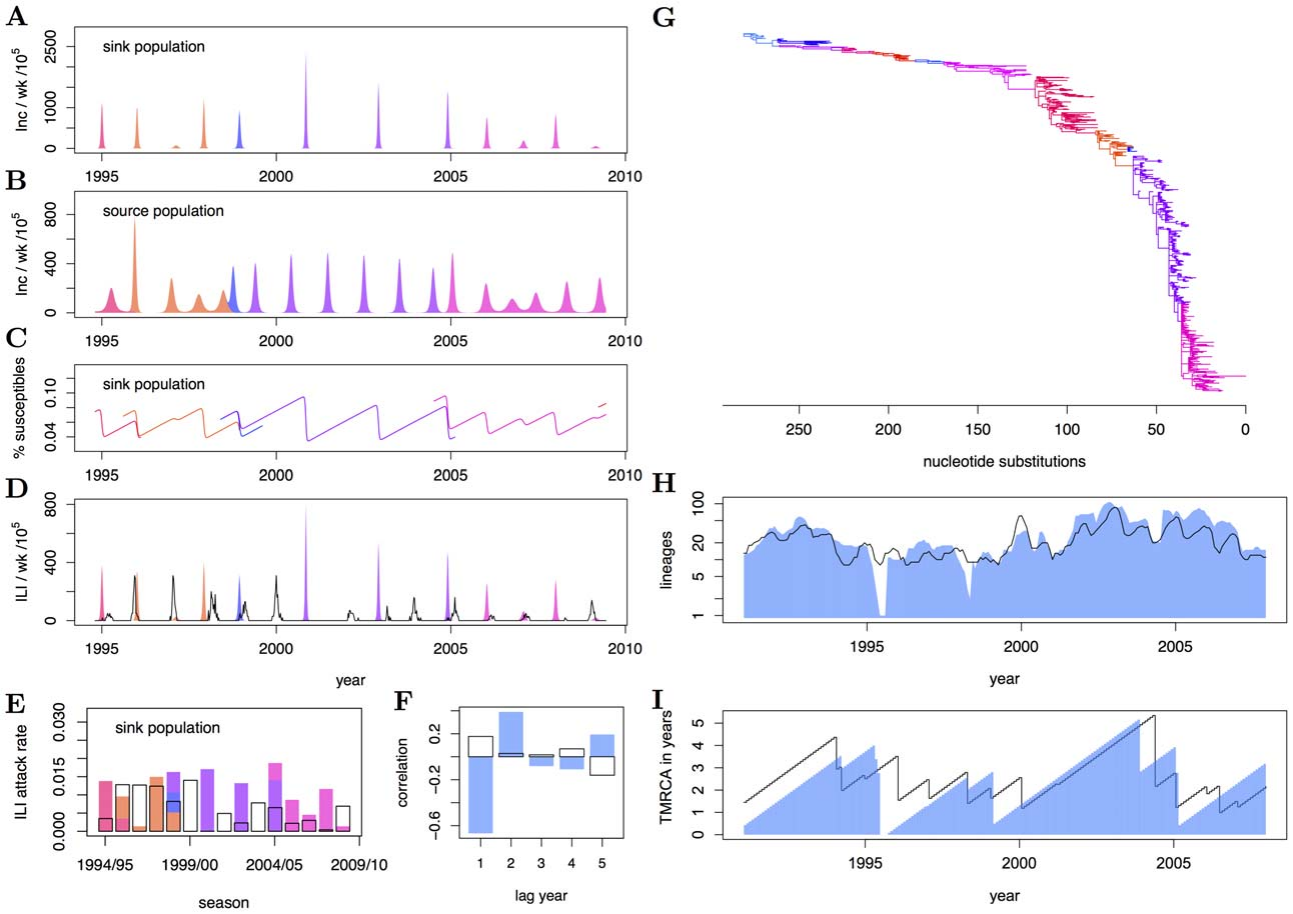
Phylodynamics arising under the fitted, spatial epochal evolution model. (A–I) Subplots are as in Figure 4. Typically, simulations under the fitted model display large infection waves and strong genetic bottlenecks at antigenic cluster invasions that are followed by refractory dynamics (A,B,H). These intrinsic dynamics result in irregular variation in case report attack rates that is well in line with H3N2 time series of several countries (E,F). Model parameters are 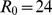, 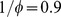, 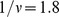, 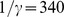, 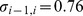, 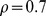, 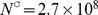, 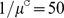, 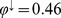, 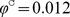, 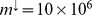, 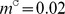, 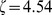, 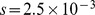, 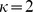, 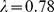, and the intrinsic dynamics are generally less pronounced when 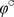 is higher; see Text S1.

We could fit and assess the epochal evolution model against summaries of H3N2 surveillance and sequence data with ABC (Figure 5 and Tables 2–3). Partial cross-immunity between mother-daughter antigenic clusters is relatively weak (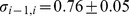), leading to abrupt changes in herd immunity and weak competition between clusters because few susceptibles are cross-depleted (see also Figure 6). In contrast to the SEIRS model, the fitted epochal evolution model excites irregular viral dynamics and reproduces the limited diversity and the small TMRCA's of H3N2's HA phylogeny (Figures 3G–I versus Figures 5I–K). This enabled us to use tighter weighting schemes for several summaries under the epochal evolution model (see Table 1). The choice of summaries influences parameter estimates in a similar manner as for the SEIRS model, see Text S1.

**Table 3:** Goodness of fit to summaries of H3N2 surveillance data and phylogenies.

summary	mean 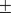 std. dev., 95% conf. interval of			
	posterior density under the SEIRS model	comments	posterior density under the epochal evolution model	comments
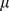 *-attack*	−0.67 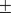 0.42, [−1.23, 0.22]	encompassing values for all countries	−0.54 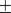 0.45, [−1.18, 0.24]	encompassing values for all countries
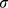 *-attack*	−0.27 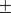 0.27, [−0.65, 0.18]	encompassing values for all countries	−0.29 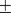 0.2, [−0.67, 0.24]	encompassing values for all countries
*explosiveness*	−0.12 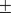 0.14, [−0.42, 0.09]	explosiveness of Dutch data not matched well	−0.06 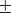 0.23, [−0.48, 0.27]	encompassing values for all countries
*correlation*	−0.84 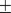 0.08, [−0.85,−0.79]	inconsistent	−0.15 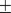 0.25, [−0.48,0.16]	encompassing values for all countries
*pop-attack*	0.03 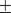 0.02, [−0.04, 0.05]	consistent	0.19 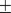 0.04, [0.1,0.17]	inconsistent in conflict with *TMRCA*
*divergence*	0.06 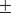 0.15, [−0.12, 0.34]	consistent	0 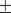 0.12, [−0.18, 0.18]	consistent
*diversity*	−0.43 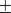 0.12, [−0.59, −0.21]	inconsistent by a factor 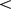 2	−0.08 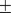 0.17, [−0.33,0.23]	consistent
*lineages*	−1.2 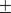 0.07, [−1.29, −1.08]	inconsistent by a factor 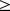 2	−0.48 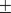 0.18, [−0.2, −0.73]	inconsistent by a factor 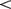 2
*TMRCA*	−0.73 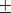 0.19, [−1.04, −0.41]	inconsistent by a factor 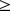 2	−0.06 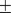 0.19, [−0.35, 0.27]	consistent
*nclust*	-		−0.03 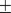 0.15, [−0.24, 0.2]	consistent

#### Goodness of fit

In the absence of strong seasonal forcing in the source population, infrequent cluster invasions often excite large invasion waves and refractory oscillations [32], as well as pronounced genetic bottlenecks that are inconsistent with the HA phylogeny, see Figure 6. More importantly, when the epochal evolution model is fitted to H3N2's narrow phylogeny, ABC reveals that aspects of disease incidence cannot be quantitatively reproduced at the same time. In particular, the estimated average duration of intra-cluster immunity is 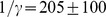 years, which in turn implies a mean posterior 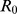 of 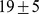 in order to reproduce H3N2's explosiveness; see Figures 5G,H. The effect of such high values of 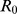 is hard to discern on interpandemic case report data without much stronger assumptions on the reporting rate than in this study. However, an 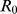 around 20 implies long term population level attack rates well below 10%, which is not compatible with epidemiological estimates (see Figure 5L and [2]). To match aspects of H3N2's HA phylogeny, unrealistically low *pop-attack* rates are further compensated by high 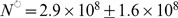. If strong seasonal forcing is assumed in the source population (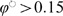 but see [42]), the epochal evolution model produces a much better fit in line with previous work [7] (see Text S1).

#### Variable selection

Finally, we identify significant levels of unexplained selection pressures in the HA phylogeny under the epochal evolution model. While the mean posterior residual selection parameter 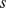 is smaller than under the SEIRS model, the posterior probability of including 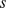 is still 1. Typically, small 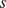 are confounded with the corresponding inclusion probability because 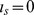 or 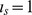 and small 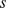 are almost equally likely [28]. Under both models, the inclusion probability is unambiguously estimated, indicating that the estimated residual selection parameter is too large to be ignored and that selection occurs not only between antigenic clusters but also within them.

## Discussion

### ABC for phylodynamic inference and model assessment

Fitting mechanistic models to infectious disease dynamics of RNA viruses that may escape immunity is notoriously difficult, and key epidemiological parameters such as 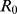 can be estimated only under tacit assumptions from incidence time series [18], [19], [43]. Currently, alternative statistical synthesis approaches are explored to harness the information in complementary data sources [25], [44], [45]. Considering summaries of interpandemic H3N2 sequence and surveillance data, we show here that ABC can be used to fit and assess complex phylodynamic models which describe how evolutionary and ecological processes of the influenza virus may interact. Key phylodynamic parameters could be estimated under relatively weak assumptions (Table 2), and ABC diagnosed readily if and in which direction the two considered models deviate from all the available data taken together.

Phylodynamic parameter inference and goodness-of-fit analyses rely critically on the possibility to combine epidemiological and molecular genetic data. In particular, H3N2 case report data were not sufficient to disentangle the reporting rate from epidemiological parameters, and measures of sequence divergence and diversity were not sufficient to separate the population size from evolutionary parameters. To the extent that other RNA viruses are characterized by different phylodynamic behavior, different sets of summaries must be identified in each case to replace likelihood calculations.

ABC relates evolutionary and epidemiological data mechanistically through an evolving dynamic system and thereby allows us to investigate empirical phylodynamic hypotheses more directly than is possible with other statistical data synthesis approaches [44], [45]. Whenever the evolution and ecology of the virus are inseparably linked [1], case report and phylogenetic summaries are co-dependent. In general, this reduces the degrees of freedom of a phylodynamic model in reproducing features of both types of data simultaneously, and may reveal model inconsistencies. For example, the fitted epochal evolution model could not reproduce the TMRCA's and the population attack rates at the same time (Figure 5L).

The reported parameter estimates and summary errors are derived by conditioning only on the phylodynamic summaries and weighting schemes described in Table 1. ABC is sensitive to the chosen summary statistics and the tolerances 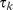 since they determine how the prior 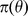 is re-weighted in light of the presented evidence (see for example Table S3 and Figure S4 in Text S1) [26]. Here, we chose broad enough tolerances 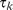 such that the weighting schemes are robust to differences in surveillance time series from the Netherlands, France and the US. This approach seems appropriate to avoid overfitting in the context of the limitations of syndromic influenza surveillance, but may be less suited in the analysis of other viral infectious diseases. Figures 3 and 5 illustrate that the resulting dimension reduction regularizes the underlying, intractable likelihood into a smooth, yet well-defined surface such that key phylodynamic model parameters are identifieable and goodness-of-fit can be characterized. It remains unclear to what degree the use of sufficient statistics or the full historical data would be desirable. Indeed, when infectious pathogens escape immunity, the likelihood surface can be especially complex [18], [43]. Likelihood-based inference is then sensitive to small changes in the complete historical data [46], [47], which can be problematic when the reported incidence time series or viral phylogeny is itself subject to considerable uncertainty and/or bias [2], [48].

### Application to H3N2 phylodynamics

We used ABC to fit mechanistic phylodynamic models of interpandemic influenza A(H3N2) to summaries of surveillance data from the Netherlands and sequence data from Northern Europe. Influenza is a globally circulating virus, and the mechanistic models considered must account for the replenishment of genetic variants from outside Northern Europe in order to reproduce features of influenza's phylogeny. In contrast, semi- or non-parametric models of population dynamics that are used in coalescent methods do not necessarily require this layer of spatial complexity [9], [20]. Here, the mechanistic structure of Eqns. (5) constrains the set of possible phylodynamic trajectories in such a way that influenza's global disease dynamics must be explicitly accounted for. Put more generally, the quantitative features of H3N2 sequence and incidence data contain sufficient information to determine at least some basic aspects of phylodynamic process models statistically.

The two models we analyzed show clear limitations in their ability to replicate features of H3N2 sequence and surveillance data simultaneously, and the ABC error diagnostics give some indication how these models could be refined (Figures 3 and 5). For example, the phylogenies generated under the SEIRS and the epochal evolution models have, across time, more lineages than the observed HA phylogeny (Table 3). One possible explanation is that localized extinctions may not occur sufficiently often under the re-seeding source-sink framework, suggesting that models with more detailed population structure, either in space or by age, may result in thinner phylogenies. Accounting for these types of population structure can be critical for understanding viral phylodynamics; here we showed that the fit of the epochal evolution model to both sequence and incidence data depends critically on the assumed spatial model structure and the associated φ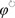 (see Text S1).

The SEIRS model could not generate the irregularity in observed incidence data. In comparison, our analysis of the epochal evolution model demonstrates that epochal evolutionary processes can easily excite irregular between-season dynamics that match observed data (see Figures 5I and S18 in Text S1). Since the virus is known to be under intense immune selection [2], it seems plausible that antigenic evolution is an important co-factor in explaining influenza's irregular seasonality in temperate regions [49].

Several alternative models have been proposed to reproduce H3N2's narrow HA phylogeny. Here, we identified an additional, testable constraint for these models on surveillance data, that arises through the phylodynamic interactions in [Disp-formula pcbi.1002835.e085] [Disp-formula pcbi.1002835.e086] [Disp-formula pcbi.1002835.e087] Eqns. (5). The cluster-specific duration of immunity 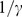 must be sufficiently long to avoid deep phylogenetic branching. If the fitted values of 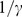 and 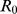 are correlated, this in turn implies a characteristic range of population level attack rates that can be tested against available data as in Figure 5L. In particular, the duration of immunity can be lower if the time between replacement events is shorter. Thus, while H3N2's limited standing genetic diversity provides information on the strength of immune interactions between H3N2 antigenic variants, this second constraint may help identify the tempo of antigenic evolution.

For the epochal evolution model with source-sink migration dynamics, the average simulated waiting time is 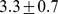 years from the emergence of the current antigenic cluster to the next successfully invading offspring antigenic cluster, and this implies phylodynamics that are inconsistent with the molecular genetic and epidemiological summaries in Table 1 taken together. More frequent and more gradual transitions between antigenic variants that are smaller than H3N2's antigenic clusters would allow for lower estimates of 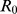 that are more in line with observed population level attack rates, break weaker refractory oscillations in their onset, and might also provide sufficient, continual selection pressures to explain the fast divergence in H3N2's HA phylogeny [8]. In this case, sequence and surveillance data would point to a finer antigenic resolution than the one suggested through antigenic map analyses [31]. Alternatively, it is also possible that finer population structure, either in space or by age, could increase extinction rates and thereby allow for a narrow HA phylogeny under a broader, more realistic set of epidemiological parameters without accelerating the tempo of antigenic evolution per se.

More broadly, both types of data are now increasingly becoming available for RNA viruses [9]–[13]. This study indicates that these data, when considered simultaneously, may drastically constrain parameter space and readily expose model deficiencies, so that ABC appears as a well-suited tool to explore the phylodynamics of RNA viruses.

## Supporting Information

Text S1 Supplementary online text describing the influenza A (H3N2) sequence and surveillance data used, ABC algorithms and summary statistics, ABC analyses on simulated data and sensitivity analyses.
(PDF)

## References

[1] Grenfell BT, Pybus OG, Gog JR, Wood JLN, Daly JM, et al (2004) Unifying the epidemiological and evolutionary dynamics of pathogens. Science 303: 327–332. 14726583 10.1126/science.1090727

[2] Cox N, Subbarao K (2000) Global epidemiology of inuenza: past and present. Annual Review of Medicine 51: 407–421. 10.1146/annurev.med.51.1.407 10774473

[3] Gog JR, Grenfell BT (2002) Dynamics and selection of many-strain pathogens. Proc Natl Acad Sci USA 99: 17209–17214. 12481034 10.1073/pnas.252512799 PMC139294

[4] Ferguson NM, Galvani AP, Bush RM (2003) Ecological and immunological determinants of inuenza evolution. Nature 422: 428–433. 12660783 10.1038/nature01509

[5] Koelle K, Cobey S, Grenfell B, Pascual M (2006) Epochal Evolution Shapes the Phylodynamics of Interpandemic Inuenza A (H3N2) in Humans. Science 314: 1898–1903. 17185596 10.1126/science.1132745

[6] Gog J (2008) The impact of evolutionary constraints on inuenza dynamics. Vaccine 26: C15–C24. 18773528 10.1016/j.vaccine.2008.04.008

[7] Koelle K, Khatri P, Kamradt M, Kepler T (2010) A two-tiered model for simulating the ecological and evolutionary dynamics of rapidly evolving viruses, with an application to inuenza. Journal of The Royal Society Interface 7: 1257–1274. 10.1098/rsif.2010.0007 PMC2894885 20335193

[8] Bedford T, Rambaut A, Pascual M (2012) Canalization of the evolutionary trajectory of the human inuenza virus. BMC Biology 10 doi:10.1186/1741-7007-10-38. 10.1186/1741-7007-10-38 PMC3373370 22546494

[9] Rambaut A, Pybus OG, Nelson MI, Viboud C, Taubenberger JK, et al (2008) The genomic and epidemiological dynamics of human inuenza A virus. Nature 453: 615–619. 18418375 10.1038/nature06945 PMC2441973

[10] Siebenga J, Vennema H, Renckens B, De Bruin E, Van Der Veer B, et al (2007) Epochal evolution of GGII.4 norovirus capsid proteins from 1995 to 2006. Journal of Virology 81: 9932–9941. 17609280 10.1128/JVI.00674-07 PMC2045401

[11] Donaldson E, Lindesmith L, LoBue A, Baric R (2010) Viral shape-shifting: norovirus evasion of the human immune system. Nature Reviews Microbiology 8: 231–241. 20125087 10.1038/nrmicro2296 PMC7097584

[12] Fischer W, Ganusov V, Giorgi E, Hraber P, Keele B, et al (2010) Transmission of single HIV-1 genomes and dynamics of early immune escape revealed by ultra-deep sequencing. PLoS One 5: e12303. 20808830 10.1371/journal.pone.0012303 PMC2924888

[13] Jabara C, Jones C, Roach J, Anderson J, Swanstrom R (2011) Accurate sampling and deep sequencing of the HIV-1 protease gene using a Primer ID. Proc Natl Acad Sci USA 108: 20166–20171. 22135472 10.1073/pnas.1110064108 PMC3250168

[14] Keeling M, Rohani P (2008) Modeling infectious diseases in humans and animals. Princeton: Princeton Univ Press. 408p.

[15] Becker N, Britton T (1999) Statistical studies of infectious disease incidence. Journal of the Royal Statistical Society: Series B 61: 287–307.

[16] Ionides EL, Breto C, King AA (2006) Inference for nonlinear dynamical systems. Proc Natl Acad Sci USA 103: 18438–18443. 17121996 10.1073/pnas.0603181103 PMC3020138

[17] Andrieu C, Doucet A, Holenstein R (2010) Particle Markov chain Monte Carlo methods. Journal of the Royal Statistical Society: Series B 72: 269342.

[18] Finkenstädt B, Morton A, Rand D (2005) Modelling antigenic drift in weekly flu incidence. Statistics in Medicine 24: 3447–3461. 16217845 10.1002/sim.2196

[19] Cauchemez S, Ferguson N (2008) Likelihood-based estimation of continuous-time epidemic models from time-series data: application to measles transmission in London. Journal of the Royal Society Interface 5: 885–897. 10.1098/rsif.2007.1292 PMC2607466 18174112

[20] Pybus O, Rambaut A (2009) Evolutionary analysis of the dynamics of viral infectious disease. Nature Reviews Genetics 10: 540–550. 10.1038/nrg2583 PMC7097015 19564871

[21] Bedford T, Cobey S, Beerli P, Pascual M (2010) Global migration dynamics underlie evolution and persistence of human inuenza A (H3N2). PLoS Pathogens 6: e1000918. 20523898 10.1371/journal.ppat.1000918 PMC2877742

[22] Bahl J, Nelson M, Chan K, Chen R, Vijaykrishna D, et al (2011) Temporally structured metapopulation dynamics and persistence of inuenza A H3N2 virus in humans. Proc Natl Acad Sci U S A 108: 19359–19364. 22084096 10.1073/pnas.1109314108 PMC3228450

[23] Drummond A, Rambaut A, Shapiro B, Pybus O (2005) Bayesian coalescent inference of past population dynamics from molecular sequences. Molecular Biology and Evolution 22: 1185–1192. 15703244 10.1093/molbev/msi103

[24] Minin V, Bloomquist E, Suchard M (2008) Smooth skyride through a rough skyline: Bayesian coalescent-based inference of population dynamics. Molecular Biology and Evolution 25: 1459–1471. 18408232 10.1093/molbev/msn090 PMC3302198

[25] Rasmussen D, Ratmann O, Koelle K (2011) Inference for nonlinear epidemiological models using genealogies and time series. PLoS Computational Biology 7: e1002136. 21901082 10.1371/journal.pcbi.1002136 PMC3161897

[26] Marin JM, Pudlo P, Robert C, Ryder R (2011) Approximate Bayesian computational methods. Statistics and Computing In press.

[27] Ratmann O, Andrieu C, Wiuf C, Richardson S (2009) Model criticism based on likelihood-free inference, with an application to protein network evolution. Proc Natl Acad Sci U S A 106: 10576–10581. 19525398 10.1073/pnas.0807882106 PMC2695753

[28] O'Hara R, Sillanpaa M (2009) A review of Bayesian variable selection methods: what, how and which. Bayesian Analysis 4: 85–118.

[29] Dijkstra F, Donker G, Wilbrink B, Van Gageldonk-Lafeber A, Van Der Sande M, et al (2009) Long time trends in inuenza-like illness and associated determinants in The Netherlands. Epidemiol Infect 137: 473–9. 18789176 10.1017/S095026880800126X

[30] Meijer A, Rimmelzwaan G, Dijkstra F, Donker G (2009) Actuele ontwikkelingen betre ffende inuenza; griepspotters in actie. Tijdschr Infect 4: 176–84.

[31] Smith DJ, Lapedes AS, de Jong JC, Bestebroer TM, Rimmelzwaan GF, et al (2004) Mapping the antigenic and genetic evolution of inuenza virus. Science 305: 371–376. 15218094 10.1126/science.1097211

[32] Koelle K, Kamradt M, Pascual M (2009) Understanding the dynamics of rapidly evolving pathogens through modeling the tempo of antigenic change: inuenza as a case study. Epidemics 1: 129–137. 21352760 10.1016/j.epidem.2009.05.003

[33] Russell CA, Jones TC, Barr IG, Cox NJ, Garten RJ, et al (2008) The global circulation of seasonal inuenza A (H3N2) viruses. Science 320: 340–346. 18420927 10.1126/science.1154137

[34] Marjoram P, Molitor J, Plagnol V, Tavaré S (2003) Markov Chain Monte Carlo without likelihoods. Proc Natl Acad Sci U S A 100: 15324–15328. 14663152 10.1073/pnas.0306899100 PMC307566

[35] Arinaminpathy N, Ratmann O, Koelle K, Epstein S, Prince G, et al (2012) Impact of cross-protective vaccines on epidemiological and evolutionary dynamics of inuenza, pnas (2011). Proc Natl Acad Sci U S A 109: 3173–3177. 22323589 10.1073/pnas.1113342109 PMC3286944

[36] Ferguson N, Cummings D, Cauchemez S, Fraser C, Riley S, et al (2005) Strategies for containing an emerging inuenza pandemic in Southeast Asia. Nature 437: 209–214. 16079797 10.1038/nature04017

[37] Pease C (1987) An evolutionary epidemiological mechanism, with applications to type a inuenza. Theoretical Population Biology 31: 422–452. 3616959 10.1016/0040-5809(87)90014-1

[38] Monto A, Koopman J, Longini I Jr (1985) Tecumseh study of illness. xiii. Inuenza infection and disease, 1976–1981. American Journal of Epidemiology 121: 811–822. 4014174 10.1093/oxfordjournals.aje.a114052

[39] Mills C, Robins J, Lipsitch M (2004) Transmissibility of 1918 pandemic inuenza. Nature 432: 904–906. 15602562 10.1038/nature03063 PMC7095078

[40] Cauchemez S, Valleron A, Boëlle P, Flahault A, Ferguson N (2008) Estimating the impact of school closure on inuenza transmission from sentinel data. Nature 452: 750–754. 18401408 10.1038/nature06732

[41] Recker M, Pybus OG, Nee S, Gupta S (2007) The generation of inuenza outbreaks by a network of host immune responses against a limited set of antigenic types. Proc Natl Acad Sci USA 104: 7711–7716. 17460037 10.1073/pnas.0702154104 PMC1855915

[42] Viboud C, Alonso W, Simonsen L (2006) Inuenza in tropical regions. PLoS medicine 3: e89. 16509764 10.1371/journal.pmed.0030089 PMC1391975

[43] Truscott J, Fraser C, Cauchemez S, Meeyai A, Hinsley W, et al (2011) Essential epidemiological mechanisms underpinning the transmission dynamics of seasonal in- uenza. Journal of The Royal Society Interface 9: 304–312. 10.1098/rsif.2011.0309 PMC3243394 21715400

[44] Ades A, Sutton A (2006) Multiparameter evidence synthesis in epidemiology and medical decision-making: current approaches. Journal of the Royal Statistical Society: Series A 169: 5–35.

[45] Birrell P, Ketsetzis G, Gay N, Cooper B, Presanis A, et al (2011) Bayesian modelling to unmask and predict inuenza A/H1N1pdm dynamics in London. Proc Natl Acad Sci U S A 108: 18238–18243. 22042838 10.1073/pnas.1103002108 PMC3215054

[46] Ramsay J, Hooker G, Campbell D, Cao J (2007) Parameter estimation for differential equations: a generalized smoothing approach. Journal of the Royal Statistical Society: Series B 69: 741–796.

[47] Wood S (2010) Statistical inference for noisy nonlinear ecological dynamic systems. Nature 466: 1102–1104. 20703226 10.1038/nature09319

[48] Stack J, Welch J, Ferrari M, Shapiro B, Grenfell B (2010) Protocols for sampling viral sequences to study epidemic dynamics. Journal of the Royal Society Interface 7: 1119–1127. 10.1098/rsif.2009.0530 PMC2880085 20147314

[49] Lipsitch M, Viboud C (2009) Inuenza seasonality: lifting the fog. Proc Natl Acad Sci U S A 106: 3645. 19276125 10.1073/pnas.0900933106 PMC2656132

