# Supplementary material for: Phylodynamic Inference and Model Assessment with Approximate Bayesian Computation: Influenza as a Case Study
Source: PLoS Comput Biol. 2012 Dec 27;8(12):e1002835. doi: 10.1371/journal.pcbi.1002835 (PMC3531293; doi:10.1371/journal.pcbi.1002835)
Supplement: Text S1 — Supplementary online text describing the influenza A (H3N2) sequence and surveillance data used, ABC algorithms and summary statistics, ABC analyses on simulated data and sensitivity analyses. (PDF) [file pcbi.1002835.s001.pdf]

Supplementary Material of

Phylodynamic inference and model assessment with Approximate Bayesian Computation: influenza as a case study

Oliver Ratmann[1,2,*], Gé Donker[3], Adam Meijer[4], Christophe Fraser[2], Katia Koelle[1,5]

1 Department of Biology, Duke University, PO Box 90338, Durham, NC 27708, USA

2 Department of Infectious Disease Epidemiology, Imperial College London, Norfolk Place, London W2 1PG, UK

3 NIVEL, Netherlands Institute for Health Services Research, P.O.Box 1568, 3500 BN Utrecht, The Netherlands

4 RIVM, National Institute for Public Health and the Environment, Centre for Infectious Disease Control, Bilthoven, the Netherlands

5 Fogarty International Center, National Institutes of Health, Bethesda, MD 20892, USA

∗ E-mail: oliver.ratmann@duke.edu

Contents

S.1 Reconstruction of H3N2 case report data 2
S.2 Reconstruction of the H3N2 haemagglutinin phylogeny 6
S.3 Two-tier MCMC algorithm 7
S.4 Calculating the effective reproductive number from model simulations 8
S.5 Choosing basic features of H3N2 sequence and incidence data 10
S.5.1 Robust summaries of H3N2 associated case report data 10
S.5.2 Inference with and without population level incidence data 10
S.5.3 Summaries of H3N2 sequence data 13
S.6 Inference and model assessment on simulated data 15
S.6.1 Parameter inference 15
S.6.2 Parameter selection 20
S.6.3 Model assessment 21
S.7 Inference with and without phylogenetic summaries 23
S.8 Inference under models without spatial substructure 25
S.9 Sensitivity analyses 26
S.10 Supplementary Figures 34

S.1 Reconstruction of H3N2 case report data

For the Netherlands, sentinel surveillance case report data from 1970 to 2009 were provided by the National Institute for Health Services Research (NIVEL), and sentinel flu virological type and subtype counts from 1994 to 2009 from the National Institute for Public Health and the Environment (RIVM) [1, 2]. Case report and virological data were collected through the Dutch sentinel general practitioner (GP) network. The sentinel GP network in the Netherlands is nationally representative by age, gender, regional distribution and population density, and the population varied between approximately 106,000 and 134,000 registered patients. ILI incidence is defined as the number of people who consulted their GP with ILI in a week divided by the population of GPs practices which reported the consultation numbers of the same week. ILI cases are defined by fever ($\geqslant 38.0°$C), sudden onset, and cough, sore throat, running nose, frontal headache, retrosternal chest pain or muscle pain (see also www.nivel.nl/peilstations). The virological data considered here correspond to patients that met ILI case definitions. Both data sets are reported by week, from Monday through Sunday, and information on age, gender and region was neglected in the present study. For France, weekly sentinel surveillance case report data from 1985 to 2009 was obtained from the 'Sentinelles' network (INSERM, UPMC) (http://www.sentiweb.frSentinelles network), and weekly virological type and subtype counts of positive ILI specimen from 1997 to 2008 were downloaded from Flunet (http://www.who.int/influenza/gisrs_laboratory/flunet/en/). For the United States, weekly nation wide case report data and virological data of positive specimen from 1997 to 2008 were obtained from the Center for Disease Control and Prevention, USA (http://www.cdc.gov/flu/weekly/fluactivitysurv.htm).

For the Netherlands, type and subtype specific case report time series from 1994 to 2009 were estimated with a regression model that relates expected weekly ILI counts to weekly virological type and subtype counts. We found that the variability in the NIVEL case report data is appropriately described under an integer count model, and there was substantial evidence for overdispersion in explaining the spread of the residuals. To exclude multiplicity effects, we used a Negative Binomial model with identity link [3]. The virological surveillance data set includes weekly counts of ILI specimen that tested negative, which allowed us to model the baseline of negative ILI case reports explicitly. Compared to using a smooth, seasonally forced baseline function as is typically done [4, 5], this approach led to slightly higher estimates of total incidence in winter seasons. This is because the baseline is not constrained to increase in winter seasons. Our initial regression model

$$\begin{aligned} y_t &\sim \text{NegBin}(\mu_t, k) \\ \mu_t &= \beta_0 + \beta_1[\text{A(H1N1)}_t] + \beta_2[\text{A(H3N2)}_t] + \beta_3[\text{B}_t] + \beta_4[\text{RSV}_t] + \beta_5[\text{Neg}_t] \end{aligned} \tag{S1}$$

consistently overestimates total ILI past 2005, see Figure S1. We found that this trend coincides with broad changes in relative sampling effort, which we defined as the number of ILI cases per virological specimen count. Figure S1B illustrates broad changes in sampling

effort with a lowess fit. To adjust for broad changes in sampling effort, we model high sampling effort in an ad-hoc fashion with a binary variable $\mathrm{S}_t^{\mathrm{h}}$ that equals one if the lowess curve in Figure S1B is above 4.5, and zero otherwise. Thus, a simple way to account for the interaction of the previous covariates with sampling effort is the regression model

$$
\begin{aligned}
y_t &\sim \mathrm{NegBin}(\mu_t, k) \\
\mu_t &= \beta_0 + \\
&\quad [\mathrm{S}_t^{\mathrm{h}}] \left(\beta_1^{\mathrm{h}}[\mathrm{A(H1N1)}_t] + \beta_2^{\mathrm{h}}[\mathrm{A(H3N2)}_t] + \beta_3^{\mathrm{h}}[\mathrm{B}_t] + \beta_4^{\mathrm{h}}[\mathrm{RSV}_t] + \beta_5^{\mathrm{h}}[\mathrm{Neg}_t]\right) + \\
&\quad [1 - \mathrm{S}_t^{\mathrm{h}}] \left(\beta_1^{\mathrm{lw}}[\mathrm{A(H1N1)}_t] + \beta_2^{\mathrm{lw}}[\mathrm{A(H3N2)}_t] + \beta_3^{\mathrm{lw}}[\mathrm{B}_t] + \beta_4^{\mathrm{lw}}[\mathrm{RSV}_t] + \beta_5^{\mathrm{lw}}[\mathrm{Neg}_t]\right).
\end{aligned}
\tag{S2}
$$

Figure S1C displays the estimated type and subtype specific incidence time series under model (S2), and Figure S1D (red) illustrates that this model explains the variation in case report data more homogeneously than model (S1). The difference $\Delta_{S1,S2}$ in the Akaike information criteria of models (S1) and (S2) is 93. Thus, accounting for broad changes in sampling effort leads to improved model predictions and improved model fit. While more refined statistical approaches to account for changes in sampling effort are possible in principle, the broad adjustment in terms of a binary categorical variable in model (S2) is sufficient for our purposes, particularly in comparison to the marked differences between estimated H3N2 incidence time series across countries.

Alternative estimation methods can have a profound effect on the magnitude and the shape of the estimated type and subtype specific time series [5]. We also compared the H3N2 time series under model (S2) to an alternative estimate derived under a standard Serfling regression model [4]. Specifically, we first reconstructed a baseline seasonal ILI time series from interannual ILI counts with the Negative Binomial Serfling regression model

$$
\begin{aligned}
y_t &\sim \mathrm{NegBin}(\mu_t, k) \\
\mu_t &= \beta_0 + \beta_1 t + \beta_2 t^2 + \beta_3 \cos(2\pi t/52) + \beta_4 \sin(2\pi t/52).
\end{aligned}
\tag{S3}
$$

Existing estimates of the timing and the duration of flu seasons were used to define the interannual period [6]. Next, we defined excess ILI as the difference between total ILI and predicted baseline ILI during epidemic periods as in [4], and took the weekly proportion of H3N2 virus counts among all specimen that tested positive in the respective week to reconstruct a weekly H3N2 excess ILI time series. Figure S2A illustrates that this excess H3N2 ILI time series is in broad agreement with the estimate under model (S2).

For France and the United States, we estimated H3N2-specific case report time series from 1997 to 2009 with the above Serfling approach. Excess ILI and H3N2 excess ILI are illustrated in Figures S2B-C for both countries. We observed large variations in relative sampling effort over time, which precluded the application of the multiple regression model (S1). The reconstructed weekly H3N2 time series across countries are compared in Figure S2D, and differ in magnitude and interannual variatiability, see Figure 1 and Table 1.

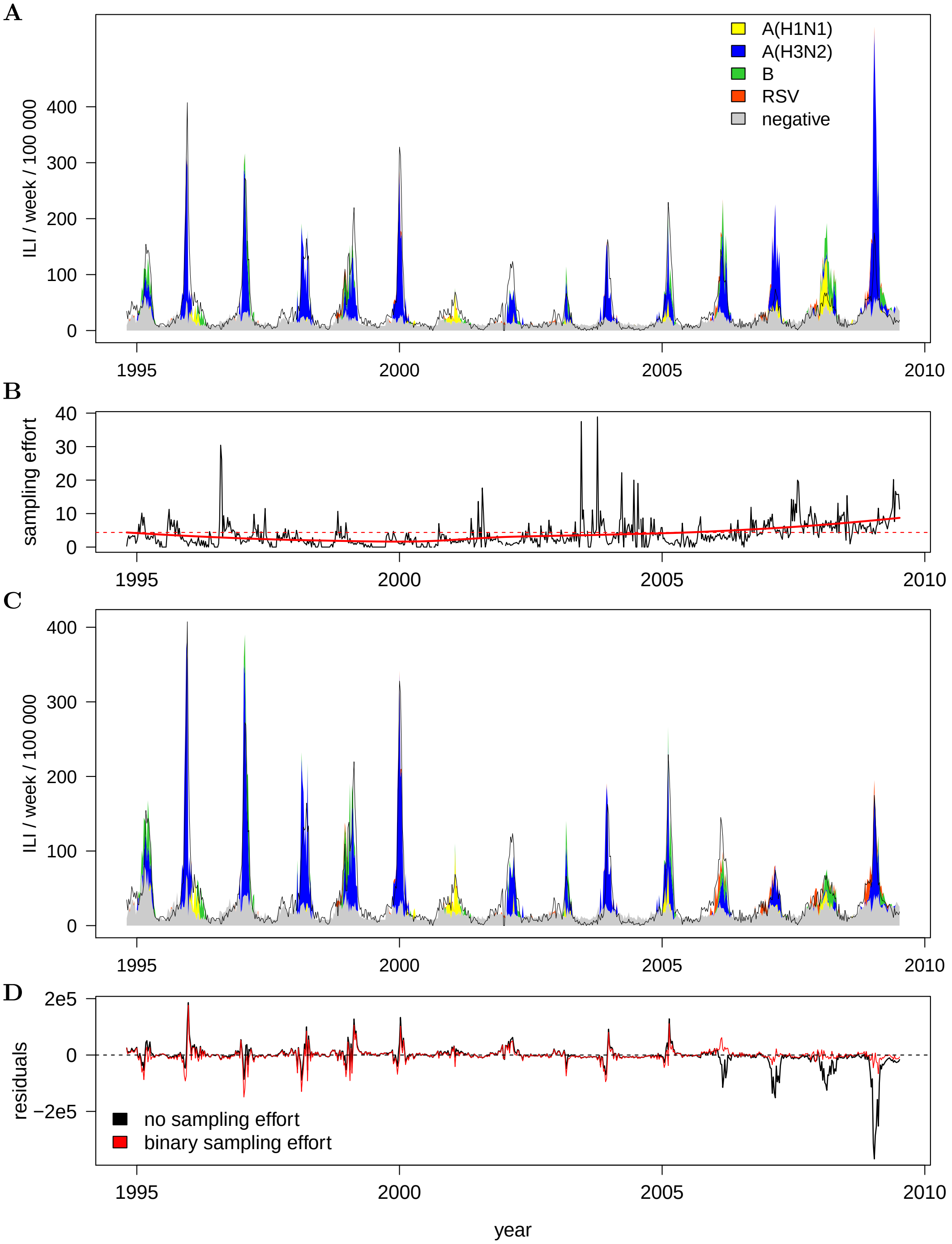

Figure S1. Estimated type and subtype specific surveillance times series in the Netherlands. (A) Estimated weekly type and subtype time series under the Negative Binomial multiple regression model (S1) with identity link. Total ILI is overlaid (black). Past 2005, total ILI is overestimated. (B) Weekly time series of relative sampling effort (see main text), and fitted smooth lowess curve (red). Past 2005, relatively more virological specimen were collected. We arbitrarily classified into low and high relative sampling effort S_t^h (red dashed line). (C) Estimated weekly type and subtype time series under the Negative Binomial multiple regression model (S2) that accounts for changes in S_t^h; same color coding as in (A). (D) Comparison of the residuals under model (S1) (black) and (S2) (red). Past 2005, model (S2) does not overestimate total ILI.

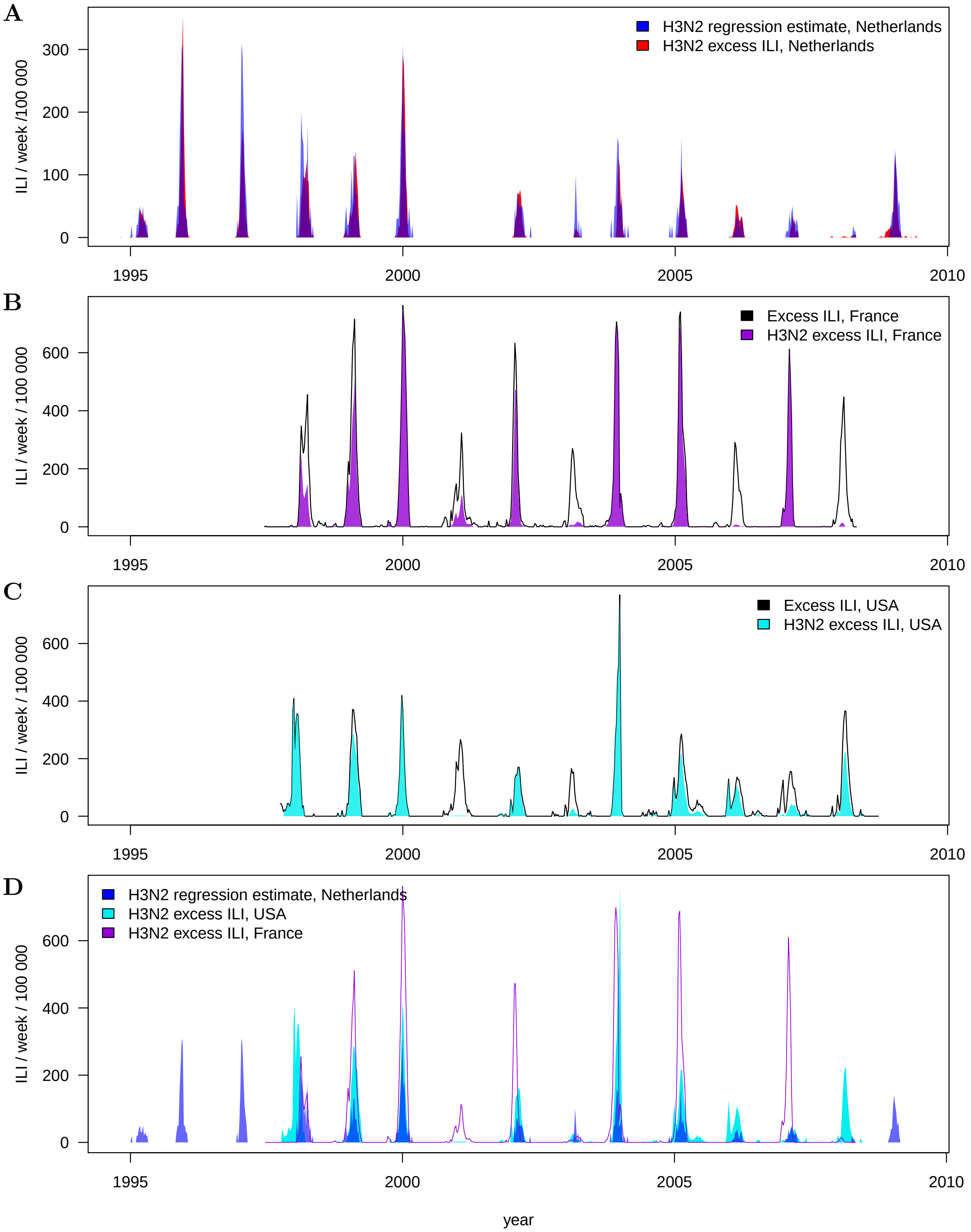

Figure S2. Comparison of H3N2 time series estimates. (A) H3N2 times series for the Netherlands, estimated with the regression model (S2) (blue) and the Serfling model (S3) (red). (B) H3N2 time series for France, estimated with the Serfling model (S3) (pink) and total excess ILI (black). (C) H3N2 time series for the USA, estimated with the Serfling model (S3) (turquoise) and total excess ILI (black). (D) The estimated H3N2 time series across countries that underlie the summaries displayed in Figure 1 and Table 1 (same color codes).

S.2 Reconstruction of the H3N2 haemagglutinin phylogeny

Sequences of the HA1 domain of the human influenza subtype H3N2 haemagglutinin gene with at least 987 nucleotides were downloaded from the Influenza Virus Resource of GenBank, and only non-lab strains of known geographic origin with at least partially specified dates between 1968 and 2009 were retained. These sequences were aligned with ClustalW v2.0.10 under default parameters, and the alignment was further manually curated to correct for obvious misplacements. A subset of 776 aligned sequences of Western European origin was taken to reconstruct the HA1 phylogeny with BEAST v1.6.1. Partially missing GenBank dates were imputed from the empirical distribution of fully specified GenBank dates, which showed a marked bias for winter seasons. Using these GenBank dates as the sampling times of the respective H3N2 strains, BEAST was run under the GTR+I+Γ nucleotide evolution model with different molecular clocks and different population demographic parameters. BEAST MCMC parameters were fine-tuned from pilot runs, and a burn-in period of 2.5 million MCMC iterations was removed. Table S1 summarizes the effect of different assumptions on population demography and molecular clocks on the estimated marginal log likelihood and the average clock rate as well as the coefficient of variation thereof. The constant clock assumes that all branches of the HA1 phylogeny evolve at the same rate, and results in relatively low clock rates. Generally higher clock rates with substantial variability among lineages are estimated with relaxed clocks, and lead to better marginal log likelihoods. Thus, we considered further only the BEAST trees inferred under relaxed molecular clocks and a piecewise constant skyline (bold in Table S1). We computed respective maximum clade credibility trees with TreeAnnotator v1.6.1, and found that the corresponding phylogenetic summaries *divergence*, *diversity*, *lineages*, *TMRCA* did not differ substantially, see Figure 1 and Table 1.

Table S1. Comparison of estimated HA1 phylogenies of Western European H3N2 strains, 1968-2009

population parameter	molecular clock	marginal log likelihood	average clock rate (/site/yr)	cv clock rate
const	const	-17040	3.5×10^{-3}	-
const	Exponential	-16740	4.5×10^{-3}	0.95
const	log normal	-16820	3.9×10^{-3}	0.67
skyline	const	-17030	3.5×10^{-3}	-
**skyline**	**Exponential**	**-16724**	$\mathbf{4.5 \times 10^{-3}}$	**0.94**
**skyline**	**log normal**	**-16800**	$\mathbf{3.8 \times 10^{-3}}$	**0.62**

For different population demographic parameters and molecular clocks, posterior mean estimates from the BEAST MCMC trees after burn-in are reported. As an alternative to a constant effective population size, a piecewise constant Bayesian skyline of 20 groups was used.

Table S2. Gelman-Rubin convergence diagnostics for the MCMC output under the SEIRS model and the epochal evolution model (Figure 3 and Figure 5 respectively)

	R_0	$1/\gamma$	ρ	s	ι_s	ζ	$N^{\circlearrowleft}$	$\varphi^{\downarrow}$	$\varphi^{\circlearrowleft}$	$m^{\downarrow}$	$m^{\circlearrowleft}$	$\sigma_{i-1,i}$	λ
Figure 3	1.02	1.01	1.01	1.02	-	1.04	1.04	1.01	1.03	1.00	1.01	-	-
Figure 5	1.13	1.02	1.02	1.07	-	1.07	1.05	1.06	1.08	1.02	1.03	1.03	1.01

S.3 Two-tier MCMC algorithm

Simple ABC rejection samplers are computationally inefficient whenever the posterior density $\pi_\tau(\theta|x)$ is markedly different from the prior density $\pi(\theta)$ [7]. We expect this situation in most phylodynamic applications. Several MCMC and sequential importance sampling algorithms are available to improve computational efficiency [8, 9]. These algorithms must be run for some time, or "burn-in", until samples from the ABC target density are generated. Phylodynamic simulations can be time consuming, particularly when the mutation rate is high. To reduce the number of simulations during burn-in, we use an MCMC sampler exactly as the one in Figure 2, but with a standard annealing scheme on the tolerances τ_k and on the variance of the proposal density that updates at acceptance [10]. In addition, a suitable choice of initial values θ^0 can improve the convergence and overall runtime of Monte Carlo algorithms. Here, we exploit the two-tier structure of phylodynamic models of the form (5) to generate initial values in a two stage process. Starting values for the parameters in the first tier (5a-5b) of a phylodynamic model are randomly chosen, and the first tier is fitted to summaries of surveillance data with algorithm mABC. Next, a set of parameter values from the posterior distribution under the summaries of the surveillance data is randomly chosen, considered fixed, and augmented by randomly chosen parameter values required in the second tier (5c-5d). Then, the second tier is fitted to the phylogenetic summaries with algorithm mABC. A set of parameter values from the posterior distribution under the phylogenetic summaries and the fixed tier 1 parameters is randomly chosen as initial value θ^0.

We applied the MCMC algorithm in Figure 2 with annealing on the tolerances τ_k and the diagonal covariance matrix of the Gaussian proposal density. Here, the annealing schemes were updated at acceptance events. Since the MCMC sampler updates only a single particle in relation to its previous value, convergence to the target density is typically reached quickly. To assess the convergence of the above algorithm, we run mABC in parallel and compare the variability of the generated Markov chains within a run to the variability across runs. The Gelman-Rubin diagnostic was computed [11], and convergence was rejected when the diagnostic exceeded 1.2 (see Table S2). The acceptance probabilities of the MCMC sampler in Figure 2 are typically below 5% and the algorithm may occasionally get stuck in the tails of the target density as in Figure 3A [12]. As further detailed in the technical report [13],

we suggest using a sequential importance sampler after convergence to quickly replenish effective sample sizes. Here, we only used the above MCMC sampler.

S.4 Calculating the effective reproductive number from model simulations

We showed that the basic reproductive number R_0 under a phylodynamic model can be substantially larger than the effective reproductive number R_{eff} when loss of immunity due to the antigenic evolution is accounted for, see Figure 3 and Figure 5. In this section, we outline how R_{eff} was back-calculated from model simulations.

We used simulated daily total population-level incidence data in the seasonally forced sink population to obtain reproductive numbers that are comparable to empirical estimates of R_{eff} of H3N2 epidemics in the Northern Hemisphere. Growth rates cannot be well estimated when incidence is low in summer seasons, and were therefore calculated only when incidence increased above a tolerance of 10% of the following peak as illustrated in Figure S3. Remaining noise was smoothed with a sliding window of two weeks as in [14], and the largest growth rate in each season was used to estimate seasonal effective reproductive numbers under the generation time distribution specified by the phylodynamic model [15]. In particular this implied that estimates of R_{eff} always exceed one. Figure S3 illustrates that typical estimates of peak seasonal R_{eff} from simulated daily population-level incidence data are fairly robust to the assumed shape of the generation time distribution and the degree of smoothing.

In the main text, we report the average of the seasonal effective reproductive numbers arising under an Exponentially distributed incubation period with a mean of 0.9 days, and an Erlang distributed infectiousness period with shape parameter 2 and a mean of 1.8 days [15].

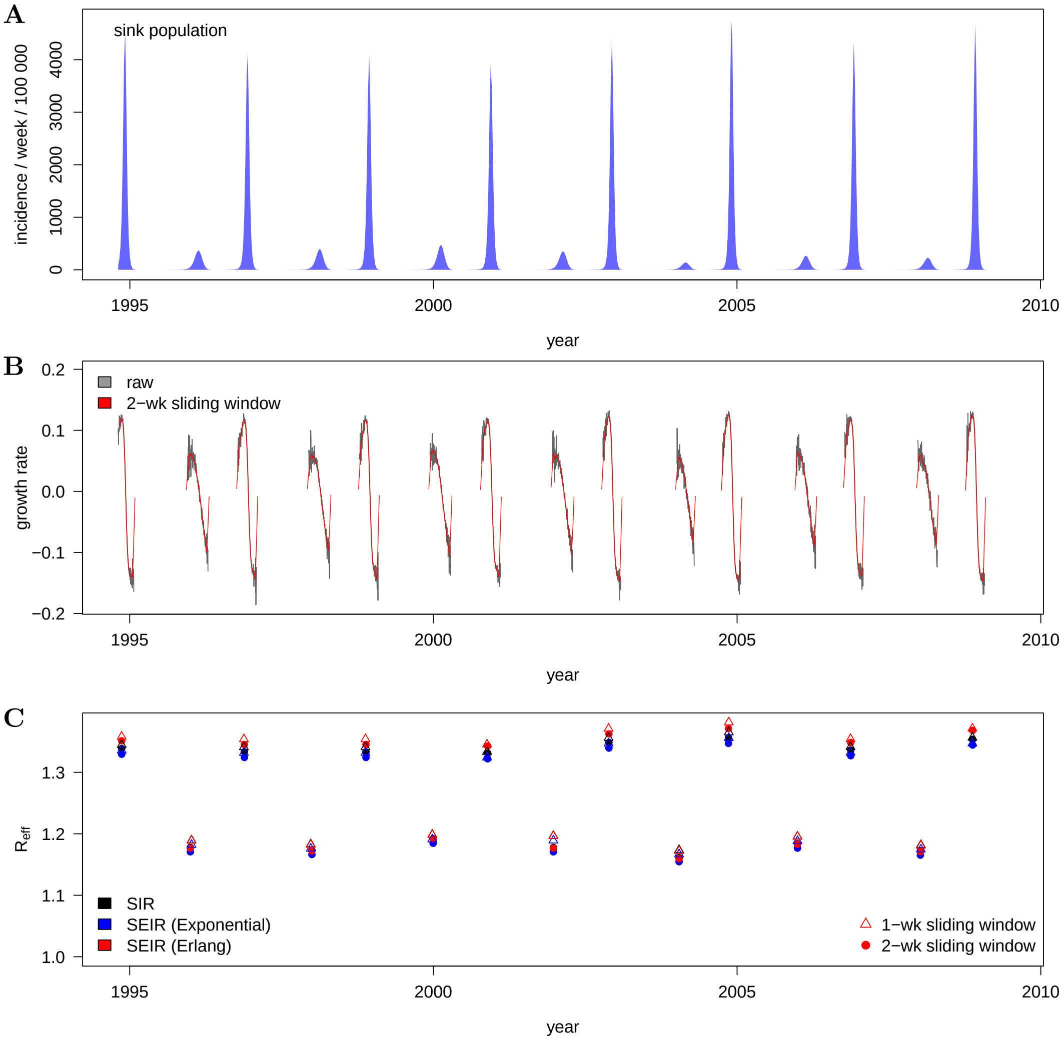

Figure S3. Typical estimates of peak seasonal R_{eff} under the fitted SEIRS model. (A) Simulated incidence in the sink population under $R_0 = 3$, $1/\phi = 0.9$, $1/\nu = 1.8$, $1/\gamma = 8.5$, $N^{\circlearrowleft} = 1.2 \times 10^8$, $1/\mu^{\circlearrowleft} = 50$, $\varphi^{\downarrow} = 0.4$, $\varphi^{\circlearrowleft} = 0.01$, $m^{\downarrow} = 7.7 \times 10^6$, $m^{\circlearrowleft} = 0.04$. (B) Corresponding growth rate in winter seasons (grey), and two-week sliding window (red). (C) Estimated peak seasonal R_{eff} under a fixed generation time of 2.8 days, and different generation time distributions and smoothing intervals. For the susceptible-infected-recovered (SIR) model, generation time follows an Exponential distribution. For the susceptible-exposed-infected-recovered (SEIR) model, the mean incubation period was set to 0.9 days, and the average time an individual remained infectious was set to 1.8 days. We considered two cases, first the infectiousness period was assumed to follow an Exponential distribution and second an Erlang distribution with shape parameter set to 2 as in the main text.

S.5 Choosing basic features of H3N2 sequence and incidence data

S.5.1 Robust summaries of H3N2 associated case report data

Interpandemic H3N2 case report data in Northern temperate regions are broadly characterized by explosive seasonal epidemics in winter months and substantial irregular interannual variation [1]. Particularly the magnitude of reported disease incidence varies across countries, likely reflecting differences in reporting practices and/or health-seeking behavior, and we identified summaries of H3N2 case report data that are largely insensitive to these differences (see Figure 1 and Table 1). A robust measure of the magnitude in interseasonal variation is the standard deviation in the log ratio of consecutive seasonal case report attack rates because it is based on fractional temporal information in cumulated case report data (*σ-attack*). To capture the irregularity in interseasonal variation, we compute the autocorrelation in case report peaks for the first few lag years (*correlation*). Autocorrelation coefficients at the same lag year can vary largely, also because the H3N2 time series is relatively short. We only use the *correlation* to penalize against strong periodic model behavior; see Table 1. Among other possibilities, the mean seasonal attack rate of reported cases (*μ-attack*) describes disease magnitude well and is informative of the country-specific reporting rate ρ. Finally, we quantify the explosiveness of winter epidemics with their average duration at half their peak size (*explosiveness*) to escape substantial uncertainty in identifying the onset and end of H3N2 seasons.

S.5.2 Inference with and without population level incidence data

In general, surveillance time series data alone is not sufficient to estimate both unknown reporting rates and the transmission parameters R_0, $1/\gamma$ of a communicable disease that experiences waning immunity [16]. Here, we show that relatively vague information on population-level incidence data is sufficient to disentangle the reporting rate from the transmission parameters when the generation time distribution is assumed known. For H3N2, we consider the largest seasonal population-level attack rate in the sink population (*pop-attack*). This summary is less sensitive to differences in periodic model behavior than the average population-level attack rate, and was favored in our analysis because the fitted SEIRS model showed strong periodicity.

For simplicity, we consider the first tier of the SEIRS model and fit it to the epidemiological summaries *μ-attack*, *σ-attack*, *correlation*, *explosiveness* and *pop-attack* as described in Table 1 under different specifications of the weighting scheme for *pop-attack*. We employ the same prior densities as in Table 2.

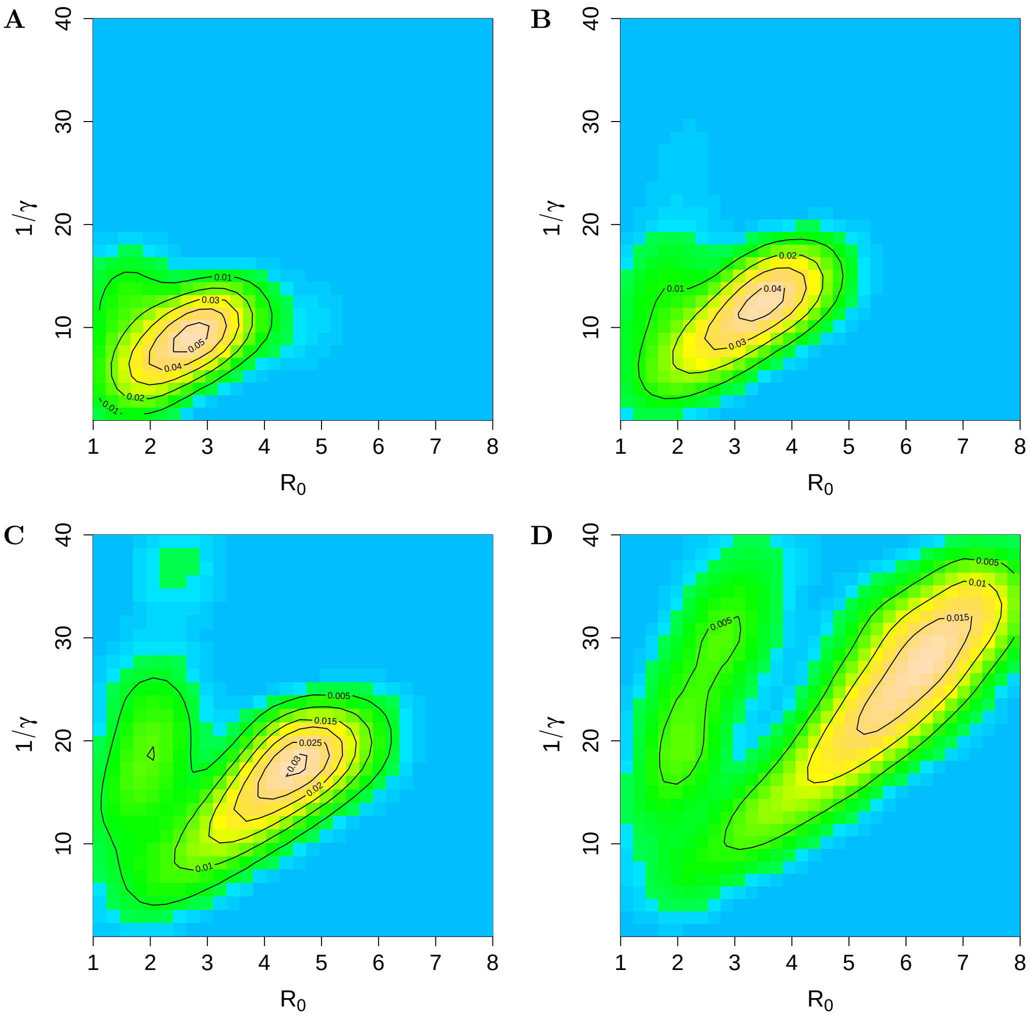

Figure S4. Parameter estimates for R_0, $1/\gamma$ of the first tier of the spatial SEIRS model under broader weighting schemes with respect to *pop-attack*. We interfaced the first tier of the SEIRS model with the epidemiological summaries described in Table 1, broadening only τ^+ of *pop-attack* from (A) 0.05 to (B) 0.08, (C) 0.1, (D) 0.13. Two-dimensional histograms in (R_0, $1/\gamma$) of the estimated ABC target density illustrate that an increasingly multimodal and broader fit is obtained when population-level incidence is allowed to differ more broadly from empirical estimates.

Table S3. Estimated epidemiological model parameters of the first tier of the SEIRS model in the absence of phylogenetic summaries

	**mean±std. dev., 95% conf. interval of posterior density based on** *pop-attack* **Indicator weighting scheme,**			
	$\tau^- = -0.1$ $\tau^+ = 0.05$	$\tau^- = -0.1$ $\tau^+ = 0.08$	$\tau^- = -0.1$ $\tau^+ = 0.1$	$\tau^- = -0.1$ $\tau^+ = 0.13$
**model parameter**				
R_0	2.46±0.80, [1.39, 3.74]	3.20±0.79, [1.46,4.44]	4.93±1.86, [1.48,7.66]	5.00±2.01, [1.95,7.78]
$R_{\mathrm{eff}}{}^{\star}$	1.26±0.07, [1.16, 1.38]	1.25±0.05, [1.16, 1.35]	1.27±0.06, [1.18, 1.39]	1.25±0.05, [1.17, 1.34]
$1/\gamma$	9.1±2.7, [4.6, 14.0]	11.6±3.8, [5.0, 16.7]	16.3±6.4, [6.2, 29.7]	25.4±7.3, [13.0, 36]
$N^{\circlearrowleft}$	3.26±1.6, [0.74, 5.69] $\times 10^8$	3.36±1.61, [0.75, 5.71] $\times 10^8$	3.47±1.56, [0.91, 5.79] $\times 10^8$	3.12±1.52, [0.83, 5.64] $\times 10^8$
$\varphi^{\downarrow}$	0.39±0.13, [0.18, 0.59]	0.38±0.13, [0.17, 0.58]	0.40±0.12, [0.18, 0.58]	0.38±0.14, [0.16, 0.59]
$\varphi^{\circlearrowleft}$	0.009±0.01, [0, 0.02]	0.012±0.008, [0,0.02]	0.011±0.009, [0,0.02]	0.012±0.007, [0,0.02]
$m^{\downarrow}$	9.5±3.4, [3.6, 14.4] $\times 10^6$	9.7±3.4, [3.8, 14.4] $\times 10^6$	9.5±3.3, [3.9, 14.4] $\times 10^6$	9.6±3.3, [4.1, 14.6] $\times 10^6$
$m^{\circlearrowleft}$	0.05±0.03, [0.01, 0.1]	0.06±0.03, [0.01, 0.1]	0.05±0.03, [0.01, 0.1]	0.05±0.03, [0.01, 0.1]
ρ	0.16±0.08, [0.06, 0.31]	0.19±0.10, [0.07, 0.35]	0.24±0.13, [0.08, 0.47]	0.33±0.15, [0.12, 0.59]
**summary error**				
μ-attack	-0.69±0.42, [-1.24, 0.07]	-0.67±0.40, [-1.27, 0.13]	-0.69±0.43, [-1.25, 0.05]	-0.71±0.43, [-1.26, 0.13]
σ-attack	-0.38±0.38, [-0.69, -0.18]	-0.38±0.37, [-0.69, 0.16]	-0.24±0.39, [-0.68, 0.16]	-0.40±0.39, [-0.69, 0.15]
explosiveness	-0.22±0.19, [-0.55, 0.05]	-0.24±0.20, [-0.56, 0.04]	-0.24±0.22, [-0.57, 0.09]	-0.27±0.22, [-0.58, 0.09]
correlation	-0.73±0.24, [-0.85, -0.29]	-0.79±0.15, [-0.85, -0.58]	-0.67±0.31, [-0.85, 0.11]	-0.75±0.18, [-0.85, -0.39]
pop-attack	0±0.04, [-0.07, 0.05]	0.04±0.04, [-0.05, 0.08]	0.07±0.04, [-0.01, 0.10]	0.10±0.03, [0.05, 0.13]

* R_{eff} is not a model parameter and calculated from simulated incidence time series.

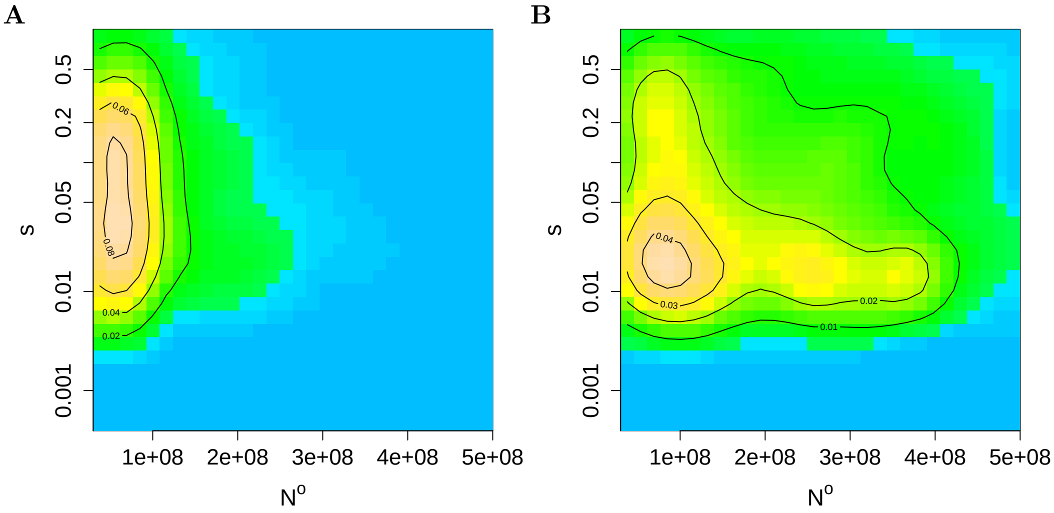

Figure S5. Parameter estimates for s, $N^{\circlearrowleft}$ of the second tier of the spatial SEIRS model with and without *lineages*. We fixed the parameters corresponding to the first tier of the SEIRS model and interfaced (s, ζ, $N^{\circlearrowleft}$) with the phylogenetic summaries described in Table 1 (A). We then repeated inference without the *lineages* (B). Two-dimensional histograms in (s, $N^{\circlearrowleft}$) of the estimated ABC target density illustrate that the source population size cannot be jointly estimated with s and ζ when *lineages* is excluded. We ran four MCMC chains in parallel, and the first tier of the SEIRS model was fixed to the four parameter sets $R_0 = 3, 2.5, 2.6, 2.7$, $1/\phi = 0.9$, $1/\nu = 1.8$, $1/\gamma = 9.6, 6.5, 8.1, 9.3$, $\rho = 0.11, 0.12, 0.07, 0.17$, $1/\mu^{\circlearrowleft} = 50$, $\varphi^{\downarrow} = 0.58, 0.31, 0.24, 0.54$, $\varphi^{\circlearrowleft} = 0.02, 0.003, 0.004, 0.002$, $m^{\downarrow} = 9.9, 7.9, 13.1, 12 \times 10^6$, $m^{\circlearrowleft} = 0.02, 0.08, 0.004, 0.009$. These parameters were chosen as described in the Methods section.

Table S3 and Figure S4 illustrate how the transmission parameters and the reporting rate of the fitted epidemiological model change with a broader *pop-attack* weighting scheme. The 95% confidence intervals of R_0, $1/\gamma$ and ρ increase as the *pop-attack* weighting scheme discriminates less against small maximum population-level attack rates, and the joint posterior density of the transmission parameters R_0, $1/\gamma$ turns increasingly irregular. The tolerances $\tau^- = -0.1$ and $\tau^+ = 0.05$ in Figure S4A correspond to largest population-level attack rates between 15-30%, which is well in line with epidemiological estimates of the population-level attack rate of influenza H3N2 in the Northern Hemisphere [17]. Larger τ^+ allow for population-level attack rates that are known to be too low, and available data do not support to choose smaller τ^+ to the best of our knowledge.

S.5.3 Summaries of H3N2 sequence data

Based on the estimated HA phylogeny, we initially summarized H3N2's evolution with its fast divergence and its limited average genetic diversity across seasons (see Figure 1 and Table 1). Specifically, divergence is captured as the slope through the number of nucleotide substitutions of sampled sequences to the founder sequence across time (*divergence*). To

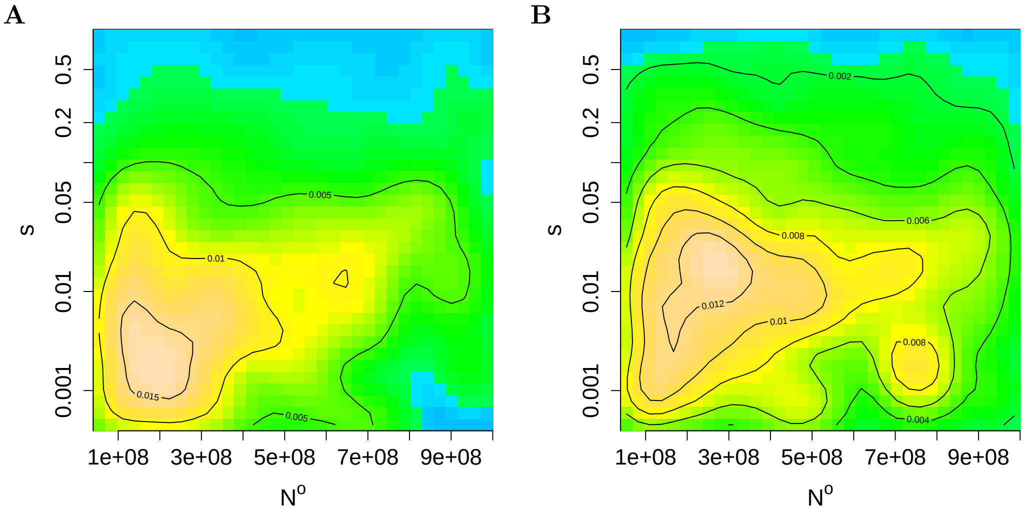

Figure S6. Parameter estimates for s, $N^{\circlearrowright}$ of the second tier of the spatial epochal evolution model with and without *lineages*. We fixed the parameters corresponding to the first tier of the epochal evolution model and interfaced (s, ζ, $N^{\circlearrowright}$) with the phylogenetic summaries as detailed in Table 1 (A). We then repeated inference without the *lineages* (B). Two-dimensional histograms in (s, $N^{\circlearrowright}$) of the estimated ABC target density illustrate that the source population size is broader when *lineages* is not included. We ran four MCMC chains in parallel, and the first tier of the epochal evolution model was fixed to the four parameter sets $R_0 = 22, 14, 20, 24$, $1/\phi = 0.9$, $1/\nu = 1.8$, $1/\gamma = 274, 276, 375, 377$, $\rho = 0.42, 0.61, 0.72, 0.86$, $1/\mu^{\circlearrowright} = 50$, $\varphi^{\downarrow} = 0.12, 0.48, 0.32, 0.22$, $\varphi^{\circlearrowright} = 0.02, 0.01, 0.02, 0.01$, $m^{\downarrow} = 3.9, 9.3, 4.9, 12.5 \times 10^6$, $m^{\circlearrowright} = 0.02, 0.04, 0.07, 0.03$. These parameters were chosen as described in the Methods section.

reflect genetic diversity, we compute the average pairwise diversity of any two sequences sampled in the same season (*diversity*). The number of dated HA sequences available before 1990 is very small, so that these years effectively do not contribute to the *diversity*. To make this sampling effect more apparent, all phylogenetic summaries except the *divergence* are only computed on the period 1991-2009.

We could not simultaneously estimate the source population size, the mutation rate and the residual selection parameter from H3N2's *divergence* and *diversity* alone. Here, we show that conditioning also on the *lineages* of the HA phylogeny is sufficient to estimate $N^{\circlearrowright}$, ζ and s. To this end, we interfaced these parameters with the phylogenetic summaries as described in Table 1 under both models, while keeping the epidemiological model parameters fixed. Figures S5-S6 illustrate that estimates of $N^{\circlearrowright}$ are broader when information on H3N2's *lineages* is not used. Since *pop-attack* is much lower under the fitted epochal evolution model, an overall larger source population size is required to yield a similar number of new genetic variants.

Finally, we found that the *TMRCA*'s are a useful summary to avoid simulated phylogenies with reasonable *divergence*, *diversity* and *lineages* but deep phylogenetic branch-

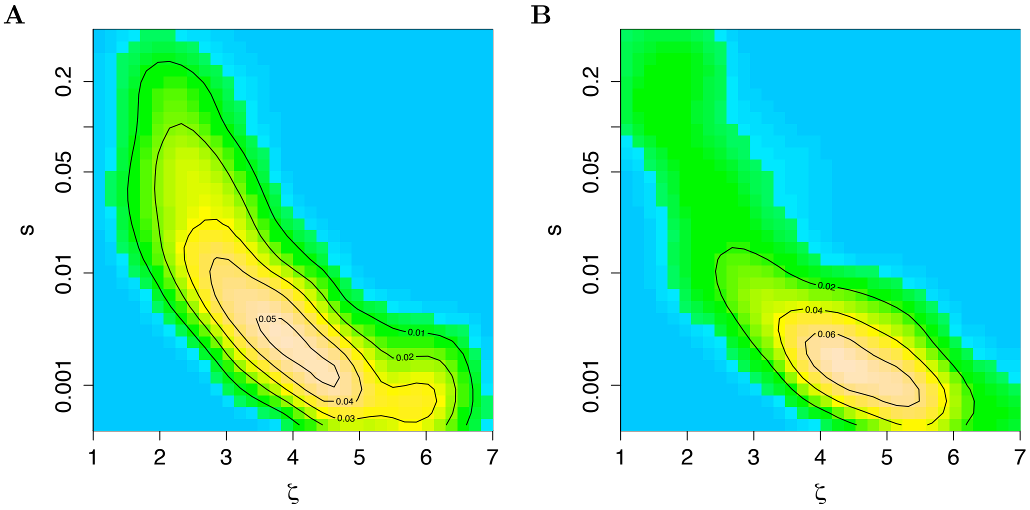

Figure S7. Parameter estimates for s, ζ of the spatial epochal evolution model with and without $TMRCA$. We repeated inference with all summaries as in Table 1 but without $TMRCA$. (A) Figure 5F for comparison. (B) Two-dimensional histogram of (s, ζ) of the estimated ABC target density without $TMRCA$.

ing. Figure S7 illustrates phylodynamic inference of the epochal evolution model with and without $TMRCA$. The selective advantage between replacing viral variants in the epochal evolution model is strong enough to limit the average pairwise diversity in simulated phylogenies, but fails to exclude deep phylogenetic branching. Thus, when inference conditions on $TMRCA$, substantial residual selection pressures must be included in the epochal evolution model. When deep phylogenetic branching is not explicitly penalized, the estimated residual selection parameter is much lower.

S.6 Inference and model assessment on simulated data

S.6.1 Parameter inference

To assess the accuracy of ABC phylodynamic inference in the context of the flu example, we first generated one data set under the SEIRS model. Model parameters were set to $R_0 = 3.5$, $1/\phi = 0.9$, $1/\nu = 1.8$, $1/\gamma = 10$, $\rho = 0.08$, $N^{\circlearrowright} = 2 \times 10^8$, $1/\mu^{\circlearrowright} = 50$, $\varphi^{\downarrow} = 0.41$, $\varphi^{\circlearrowright} = 0.006$, $m^{\downarrow} = 4 \times 10^6$, $m^{\circlearrowright} = 0.01$, $\zeta = 1.5$, $s = 0.09$, a sample of the posterior distribution under the summaries and tolerances in Table 1. Figure S8 illustrates ABC parameter estimates for this simulated data set, using summaries and tolerances as in Table 1. Estimates of R_0, $1/\gamma$, ρ were fairly broad, and broader than those obtained from inference against the real flu data set. We failed to estimate $N^{\circlearrowright}$, and the estimates of s, ζ did not correspond well to the true values. As on the real data study, $\varphi^{\downarrow}$, $\varphi^{\circlearrowright}$, $m^{\downarrow}$, $m^{\circlearrowright}$ could not be estimated. The reason for such poor parameter inference is that the summary errors against data simulated from the same model are much smaller than those computed against real data. Thus, a broader

set of parameters is now acceptable when the same tolerances are used.

Using tighter tolerances than those in Table 1, we could accurately estimate the same parameters as compared to inference on the real data (except $N^{\circlearrowleft}$). Using (the values used in the main text are added in brackets) $\tau^-_{\textit{pop-attack}} = -0.03\ (-0.1)$, $\tau^+_{\textit{pop-attack}} = 0.03\ (0.05)$, $\tau^-_{\sigma\textit{-attack}} = -0.45\ (-0.7)$, $\tau^+_{\sigma\textit{-attack}} = 0.35\ (0.35)$, $\tau^-_{\mu\textit{-attack}} = -0.45\ (-1.3)$, $\tau^+_{\mu\textit{-attack}} = 0.35\ (0.35)$, $\tau^-_{\textit{explosiveness}} = -0.35\ (-0.6)$, $\tau^+_{\textit{explosiveness}} = 0.35\ (0.35)$, we obtained more reliable estimates of R_0, $1/\gamma$, ρ as illustrated in Figure S9A. Next, we could obtain improved estimates of s, ζ with tighter tolerances on the phylogenetic summaries, $\tau^-_{\textit{divergence}} = -0.175\ (-0.4)$, $\tau^+_{\textit{divergence}} = 0.175\ (0.4)$, $\tau^-_{\textit{diversity}} = -0.2\ (-0.6)$, $\tau^+_{\textit{diversity}} = 0.2\ (0.6)$, $\tau^-_{\textit{lineages}} = -0.025\ (-1.3)$, $\tau^+_{\textit{lineages}} = 0.025\ (1.3)$, $\tau^-_{\textit{TMRCA}} = -0.75\ (-3)$, $\tau^+_{\textit{TMRCA}} = 0.75\ (3)$; see Figure S9B. We could not estimate an upper limit for $N^{\circlearrowleft}$. Tolerances must be chosen in view of the error magnitude. If the error magnitude is considerably larger on real data than on simulated data, then ABC parameter inference on real data with tolerances $\tau^\star$ calibrated on simulated data will suffer from very low acceptance rates and will thus be extremely unreliable. For example, the $\tau^\star$ of the phylogenetic summaries are smaller than the errors between the SEIRS model and the H3N2 phylogeny, so that ABC parameter inference against real data is impossible with these $\tau^\star$.

We further checked that ABC based on the summaries in Table 1 can accurately estimate a range of SEIRS model parameters with suitable tolerances, see Figure S10. In general, for higher values of R_0 smaller tolerances had to be chosen because differences in population-level attack rates decrease. For relatively large tolerances, posterior distributions were broad, but never inaccurate and reliable. We emphasise that often, it is not appropriate to set the tolerances to tight values $\tau^\star$ even when this is computationally feasible. Available data may not support the use of narrow ABC tolerances $\tau^\star$. For example, it is possible to use tighter tolerances on *pop-attack* than those in Table 1. The tolerances $\tau^-_{\textit{pop-attack}} = -0.1$, $\tau^+_{\textit{pop-attack}} = 0.05$ correspond to maximum population-level attack rates between 15=30%, which is well in line current epidemiological estimates of population-level attack rates between 10-20% [17]. Considerably tighter tolerances would result in some form of overfitting.

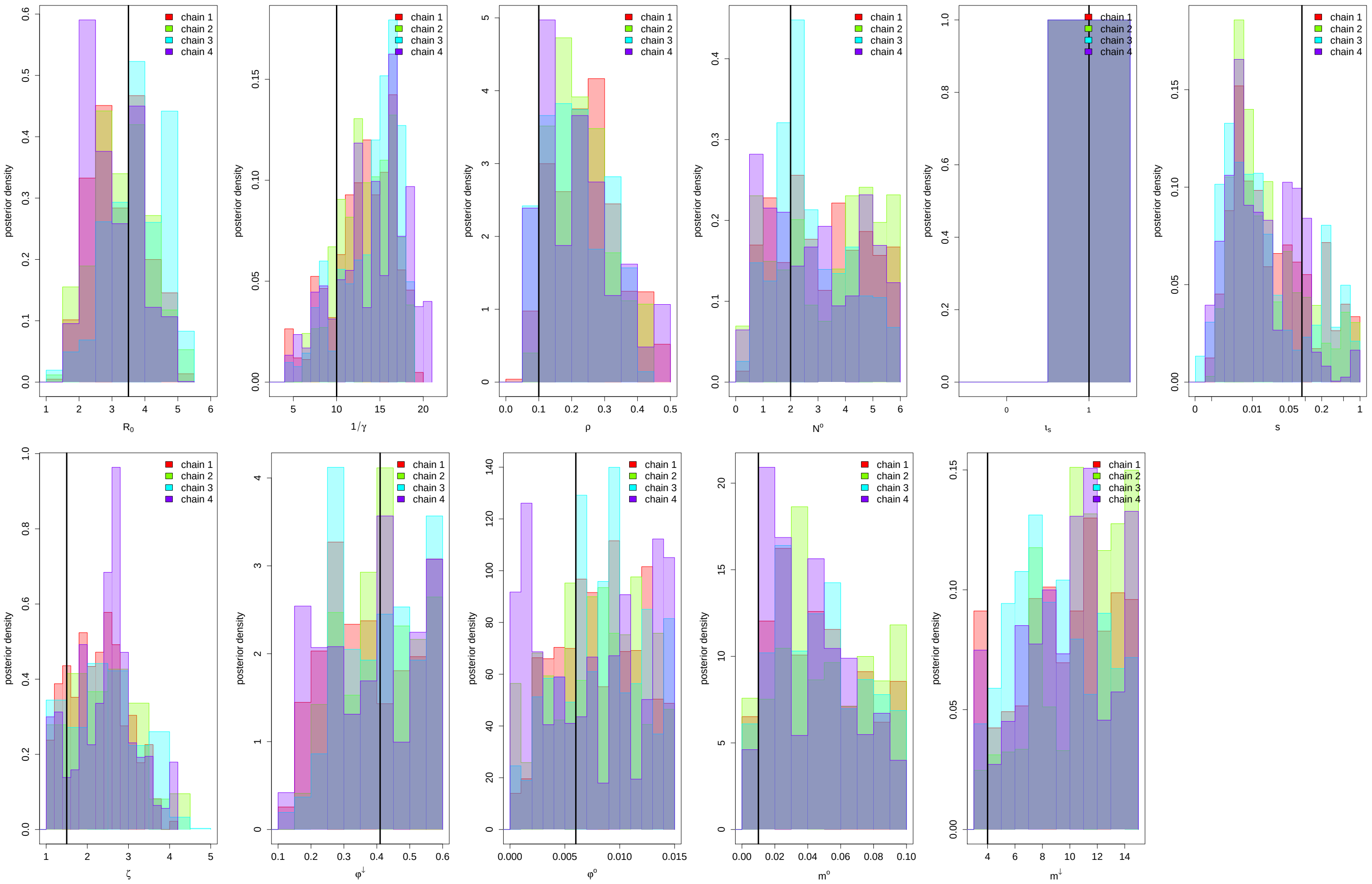

Figure S8. Parameter estimates of the spatial SEIRS model on data simulated from the spatial SEIRS model (case $R_0 = 3.5$), using summaries and tolerances as in Table 1. One-dimensional histograms of the ABC fit using the summaries and tolerances in Table 1. Four MCMC chains were started at overdispersed starting values, a burn-in period was removed and the remaining samples are shown in color for each chain. True parameters from which the data set were generated are indicated in black.

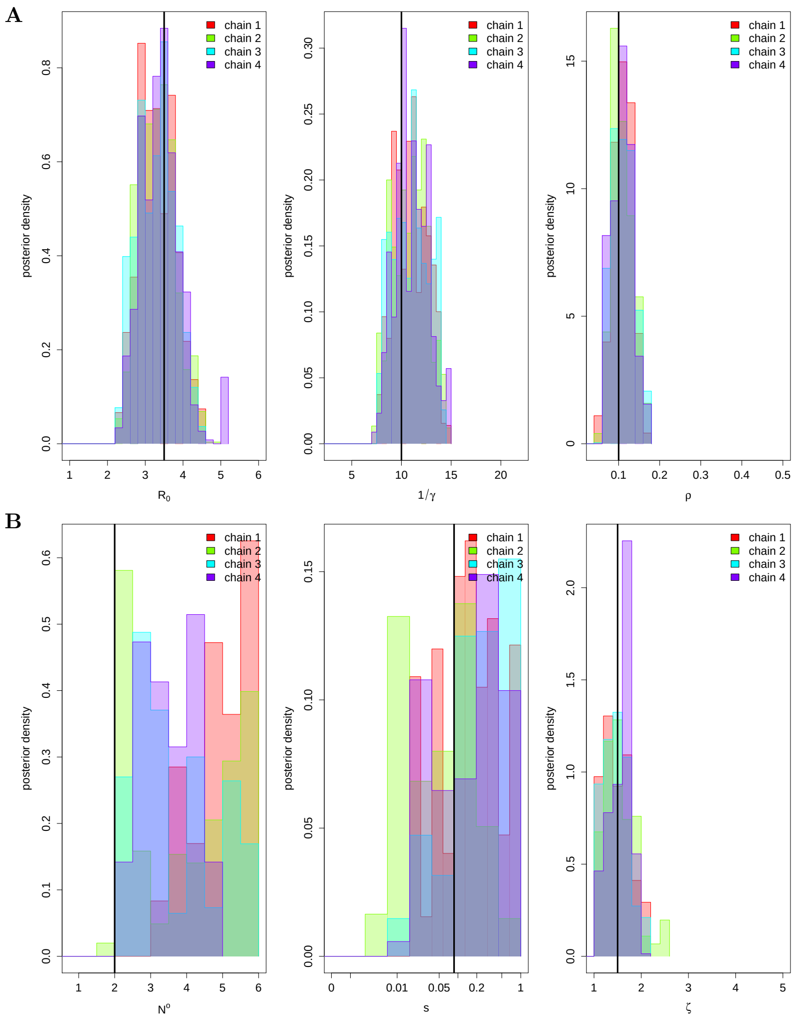

Figure S9. Parameter estimates of the spatial SEIRS model on data simulated from the spatial SEIRS model (case $R_0 = 3.5$), using tighter tolerances than in Table 1. One-dimensional histograms of the ABC fit using the summaries in Table 1 but (A) tighter tolerances on *μ-attack*, *σ-attack*, *pop-attack* and (B) tighter tolerances on *divergence*, *diversity*, *lineages*, *TMRCA* as detailed in the text. In both cases, four MCMC chains were started at overdispersed starting values, a burn-in period was removed and the remaining samples are shown in color for each chain. True parameters from which the data set were generated are indicated in black.

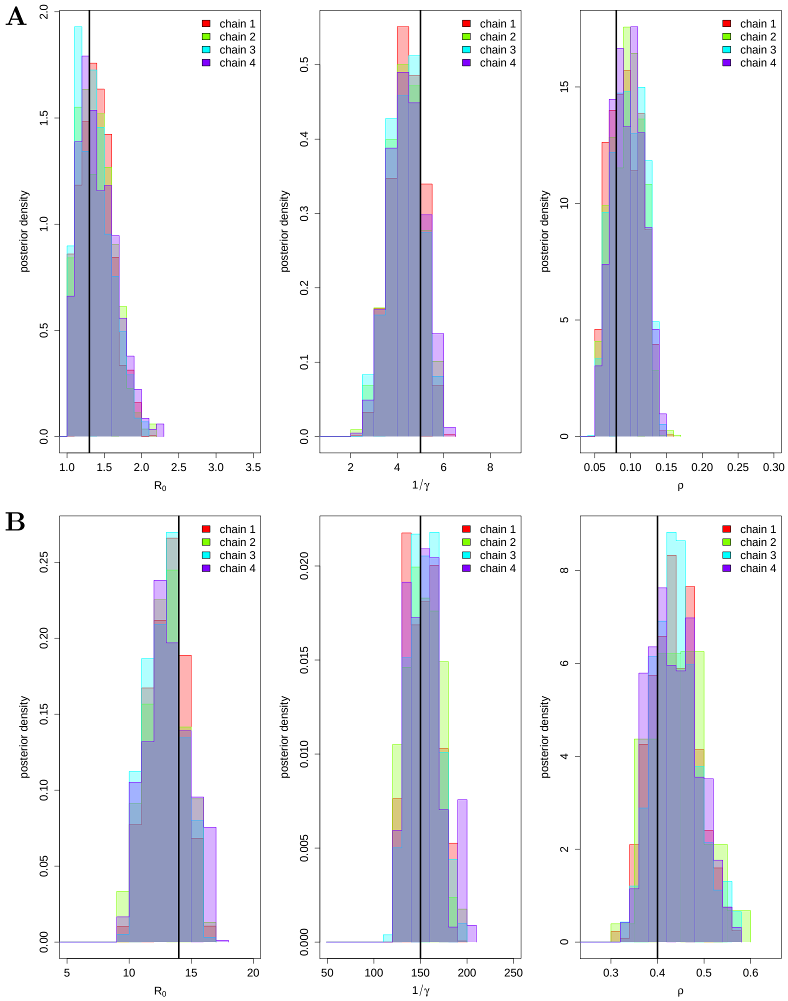

Figure S10. Parameter estimates of the spatial SEIRS model on data simulated from the spatial SEIRS model (case A: $R_0 = 1.3$, case B: $R_0 = 14$), using tighter tolerances than in Table 1. One-dimensional histograms of the ABC fit against data generated with the parameters (A) $R_0 = 1.3$, $1/\gamma = 5$, $\rho = 0.08$ and (B) $R_0 = 14$, $1/\gamma = 150$, $\rho = 0.4$ and all other parameters as before. We used the summaries in Table 1 with tolerances (A) as in Figure S9A, and (B) $\tau^+_{pop\text{-}attack} = 0.002$, $\tau^-_{pop\text{-}attack} = -0.002$, $\tau^+_{\sigma\text{-}attack} = \tau^+_{\mu\text{-}attack} = \tau^+_{\mu\text{-}attack} = \tau^+_{explosiveness} = 0.2$, $\tau^-_{\sigma\text{-}attack} = \tau^-_{\mu\text{-}attack} = \tau^-_{\mu\text{-}attack} = \tau^-_{explosiveness} = -0.2$ and all others as before. MCMC samples were generated as before, and true parameters from which the data set were generated are indicated in black.

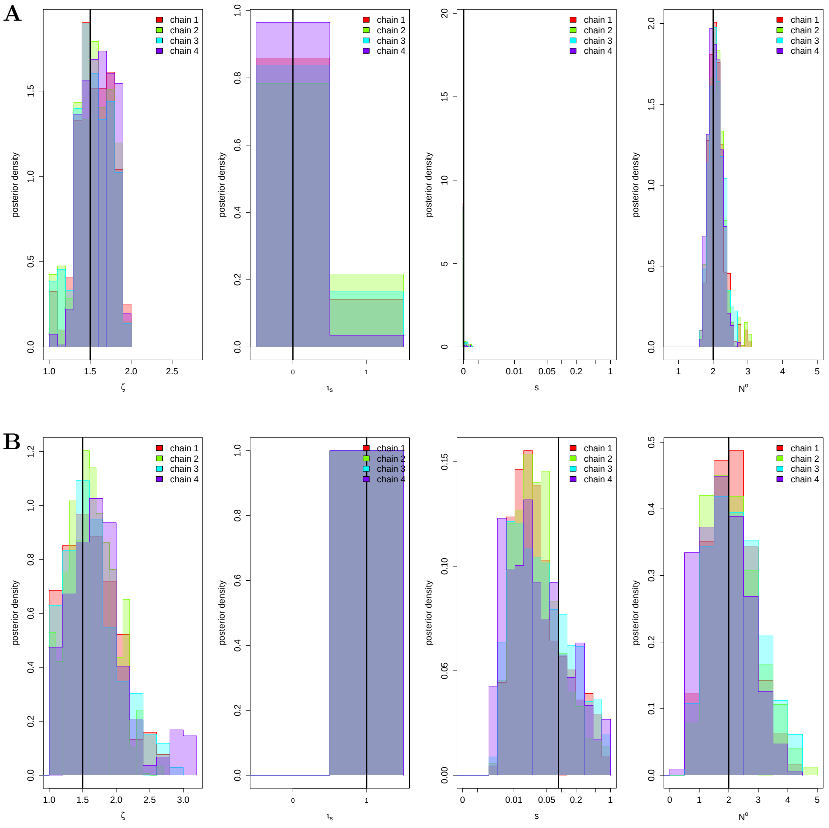

Figure S11. Accuracy in estimating the inclusion probability ι_s. One-dimensional histograms of parts of the ABC fit against simulated data, case $R_0 = 3.5$, (A) without and (B) with selection, see the text for details. Four MCMC chains were started at overdispersed starting values and the epidemiological parameters were held fixed. The first 1000 iterations were discarded and histograms of the remaining samples are shown in color for each chain. The correct value is indicated with a vertical black line.

S.6.2 Parameter selection

Next, we evaluated how reliable ABC can estimate if the selection parameter s should be included in phylodynamic models of the form (5). We generated two data sets under the SEIRS model with and without residual selection; parameters were (i) $s = 0$, (ii) $s = 0.09$, and $R_0 = 3.5$, $1/\phi = 0.9$, $1/\nu = 1.8$, $1/\gamma = 10$, $\rho = 0.08$, $N^{\circlearrowright} = 2 \times 10^8$, $1/\mu^{\circlearrowright} = 50$, $\varphi^{\downarrow} = 0.41$, $\varphi^{\circlearrowright} = 0.015$, $m^{\downarrow} = 4 \times 10^6$, $m^{\circlearrowright} = 0.01$, $\zeta = 1.5$ in both (i) and (ii). The parameters of scenario (i) correspond to the fitted SEIRS model in the main text, Figure 3. We used ABC with the same summaries as in Table 1. Prior densities were chosen as in Table 2. As before, the tolerances had to be tightened for reliable parameter inference. We used the Indicator weighting scheme (3) for all summaries and the tolerances $\tau^+_{divergence} = 0.175$, $\tau^+_{diversity} = 0.2$, $\tau^+_{lineages} = 0.035$, $\tau^+_{TMRCA} = 0.4$ and $\tau^-_k = -\tau^+_k$. Figure S11 illustrates that the inclusion probability ι_s can be reliably estimated. All other parameters are also

well estimated, although credibility intervals are substantially broader under scenario (ii). Notably, the variable selection prior penalizes large values of s so that the true values of s can be expected to be somewhat larger than estimates thereof.

S.6.3 Model assessment

In light of our results on the epochal evolution model, we first verified that this model can be accurately fitted to data generated from the same model, and that the associated summary errors are then centred around zero. Incidence and phylogenetic data were generated under the parameters $R_0 = 20$, $1/\phi = 0.9$, $1/\nu = 1.8$, $1/\gamma = 180$, $\rho = 0.75$, $\sigma = 0.75$, $N^{\circlearrowright} = 4 \times 10^8$, $1/\mu^{\circlearrowright} = 50$, $\varphi^{\downarrow} = 0.28$, $\varphi^{\circlearrowright} = 0.015$, $m^{\downarrow} = 8 \times 10^6$, $m^{\circlearrowright} = 0.05$, $\zeta = 3$, $s = 0.09$, $\kappa = 2$, $\lambda = 380$, which correspond well to the fitted epochal evolution model in the main text, Figure 5. We used ABC with the same summaries as in Table 1. Prior densities were chosen as in Table 2. As before, the tolerances had to be tightened to estimate parameters reliably, again because the summary errors were here considerably smaller for a broader set of model parameters. Figure S12A illustrates that R_0, $1/\gamma$, σ, ρ and λ could be accurately estimated under the same tolerances as in Figure S9A (which are tighter than those in Table 1). Next, again using tighter tolerances, we could obtain accurate estimates of the molecular genetic parameters (see Figure S12B). All other parameters not shown had fairly broad posterior distributions and could not be estimated. With the tolerances in Table 1, estimates of R_0 had a 95% confidence interval of $[7, 27]$ and a posterior mean at 16.9, and s, ζ could not be estimated. Figure S12C shows that all the associated summary errors plotted in Figure 5I-L are now close to zero, and Figure S12D shows that the same is true for all other summary errors, indicating that the summary errors correctly indicate goodness of fit.

Finally, we verified that ABC with the summaries in Table 1 can detect discrepancies of the SEIRS model in reproducing data generated under the epochal evolution model. Here, we used the same tolerances as in Table 1, except for *diversity*: $\tau^{-}_{diversity} = -1$, $\tau^{+}_{diversity} = 1$. Figure S13A shows the ABC summary diagnostics that we used in the main text, and Figure S13B shows all remaining ones. The *correlation*, *diversity* and *TMRCA* summary errors deviate clearly from zero, indicating that the summary errors can correctly identify model mismatch.

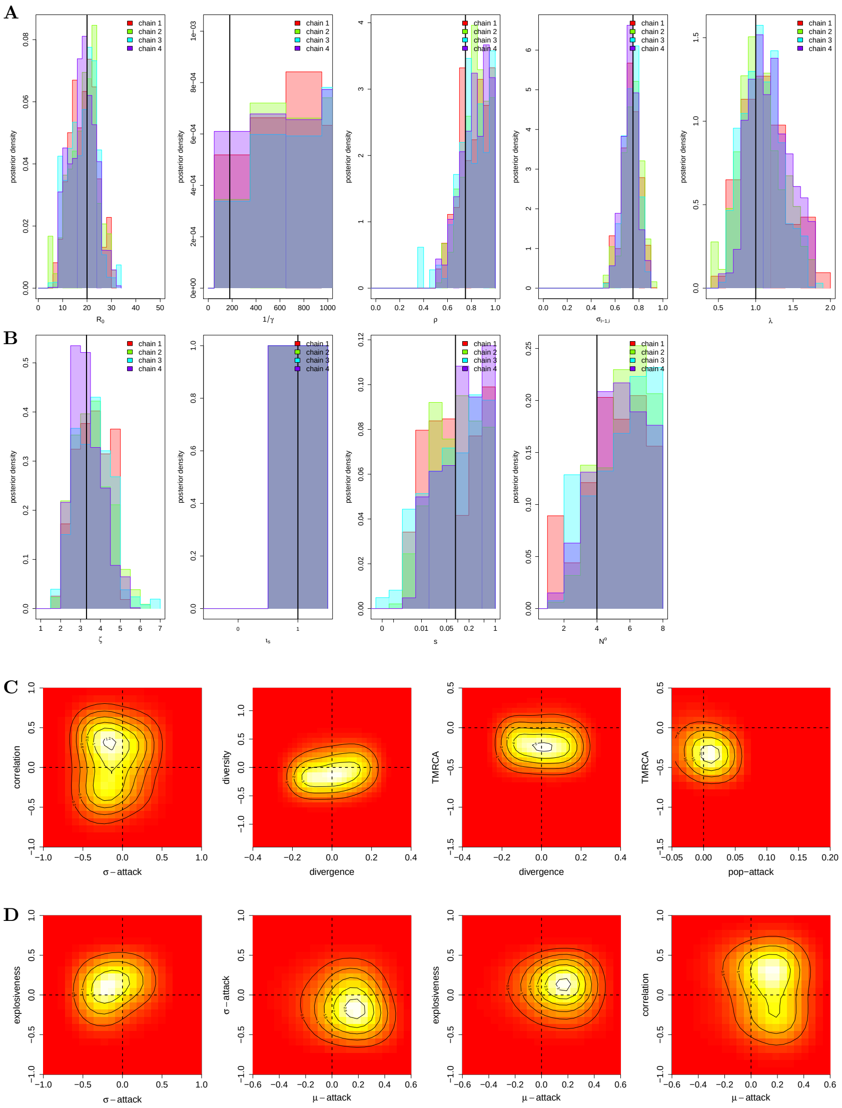

Figure S12. Parameter estimates of the spatial epochal evolution model on data simulated from the same model, using tighter tolerances than in Table 1. One-dimensional histograms of parts of the ABC fit based on (A) tighter tolerances of the epidemiological summaries (as in Figure S9A), and (B) tighter tolerances on the phylogenetic summaries $\tau^-_{divergence} = -0.2\ (-0.4)$, $\tau^+_{divergence} = 0.2\ (0.2)$, $\tau^-_{diversity} = -0.4\ (-0.6)$, $\tau^+_{diversity} = 0.4\ (0.6)$, $\tau^-_{lineages} = -0.4\ (-1.3)$, $\tau^+_{lineages} = 0.4\ (1.3)$, $\tau^-_{TMRCA} = -0.5\ (-3)$, $\tau^+_{TMRCA} = 3\ (3)$; values of Table 1 added in parentheses. MCMC samples were generated as before, and true parameters from which the data set were generated are indicated in black. (C-D) 2-D histograms of the associated summary errors.

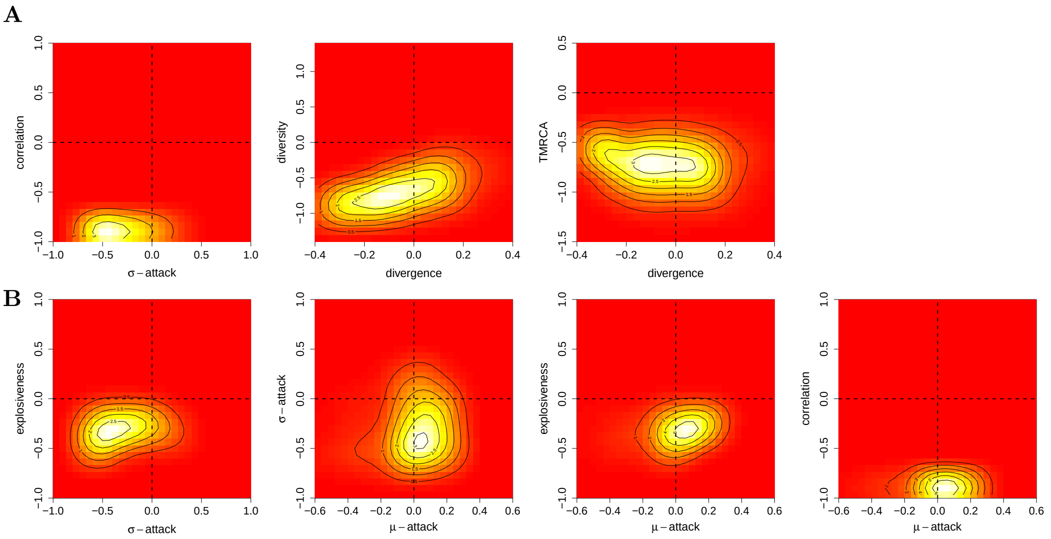

Figure S13. Accuracy in detecting discrepancies of the SEIRS model when fitted to simulations of the epochal evolution model. Two-dimensional histograms of the nine-dimensional distribution of ABC summary diagnostics that correspond to fitting the SEIRS model with the summaries in Table 1, see the text for details. (A) Summary diagnostics shown in the main text for ABC analyses on real data, and (B) diagnostics for all other summaries. Four MCMC chains were started at overdispersed parameter values, the burn-in period was removed and all remaining samples were pooled across chains to produce the histograms.

S.7 Inference with and without phylogenetic summaries

We analyzed if the phylogenetic summaries *divergence*, *diversity*, *lineages* and *TMRCA* have any effect on estimates of the epidemiological parameters of SEIRS model. To this end, we fitted the first tier of the SEIRS model to the epidemiological summaries *μ-attack*, *σ-attack*, *correlation*, *explosiveness* and *pop-attack* in Table 1, using the same prior densities as in Table 2.

Comparing Tables 2-3 to the first column in Table S3, we find that without the phylogenetic summaries, the fitted source-sink SEIRS model results in smaller basic reproductive numbers 2.46±0.80 and maximum population-level attack rates that are on average 15%±4% (3.03±0.55 and 12%±3% respectively when the full phylodynamic model is fitted). Because lower incidence results in more narrow phylogenies, this discrepancy can be explained with the addition of the phylogenetic summaries. To substantiate this explanation, we fitted the tier 1 model under a relaxed *pop-attack* weighting scheme ($\tau^+ = 0.08$), which gave a distribution of *pop-attack* posterior summary errors that is almost identical to the one obtained with the phylogenetic summaries; see the second column in Table S3. The associated R_0 is estimated at 3.2±0.79, which is very similar to the estimate obtained with the phylogenetic summaries. Thus, the imposed penalties in the weighting scheme for narrow phylogenies (see

Table 1) result in slight deviations to low maximum population-level attack rates, which implies relatively high values of R_0 when phylogenetic summaries are included. This implies that conditioning on phylogenetic summaries has an effect on parameter inference even under the SEIRS model, and the molecular genetic and epidemiological summaries are not independent of each other.

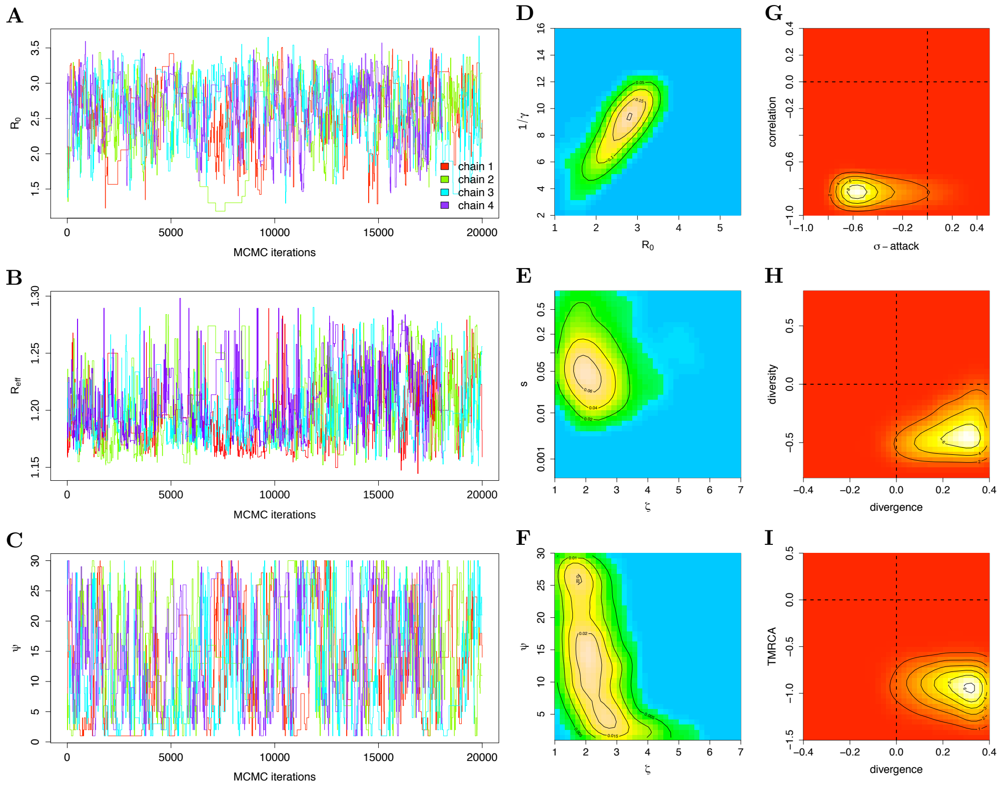

Figure S14. Phylodynamic inference and goodness-of-fit analysis of the spatially homogeneous, SEIRS model (S4). (A-C) MCMC trajectories of the estimated R_0, ψ and the calculated R_{eff} of four chains that were started at four two-tier generated seeds (see Methods). (D-F) Two-dimensional histograms of parts of the ABC fit, illustrating the correlations between the estimated parameter pairs (R_0, $1/\gamma$), (s, ζ) and (ψ,ζ). Throughout, histograms were computed from all samples across the four chains after burn-in. Color codings are separate for each subplot, with respective density values indicated in the contours. (G-I) Two-dimensional histograms of parts of the joint density of summary errors, illustrating the goodness of the fitted model with respect to the *correlation* and interannual variability of the case report data, as well as the *divergence*, *diversity* and the *TMRCA*'s of the HA phylogeny.

S.8 Inference under models without spatial substructure

Figure 3H-I illustrate that the spatial SEIRS model can reproduce the *divergence* of H3N2's HA phylogeny. To illustrate that a geographically separated source population is required to reproduce the *divergence* of H3N2's HA phylogeny, we consider here the corresponding SEIRS model in a seasonally forced, spatially homogeneous population that is, as before, calibrated to represent the Netherlands. Leaving demographic stochasticity aside, H3N2 phylodynamics are described by

$$\begin{aligned}
\frac{dS}{dt} &= \mu(N-S) - \beta_t \frac{S}{N}(I + m\frac{\hat{I}}{N}) + \gamma(N-S-E-I) \\
\frac{dE}{dt} &= \beta_t \frac{S}{N}(I + m\frac{\hat{I}}{N}) - (\mu+\phi)E \\
\frac{dI_1}{dt} &= \phi E - (\mu + 2\nu)I_1 \\
\frac{dI_2}{dt} &= 2\nu I_2 - (\mu+\nu)I_2 \\
\frac{dG_k}{dt} &= \frac{(1+s\varrho_{k0})\psi I^+}{\sum_l (1+s\varrho_{l0})G_l} G_k - \frac{\psi I^-}{\sum_l G_l} G_k - \zeta G_k,
\end{aligned} \tag{S4}$$

where all parameters are as in (5) for the sink population, and $I = I_1 + I_2$, $1/\phi$ is the average duration of incubation, m is the number of visiting infected travelers, $\hat{I}$ is the number of infected individuals at disease equilibrium, and $\psi \geqslant 1$ is an inflation factor. As we show below, this inflation factor is necessary to reproduce the *divergence* of the HA phylogeny. The second tier of (S4) thus corresponds to a first tier that is inflated by ψ.

The spatially homogeneous SEIRS model was fitted to the phylodynamic summaries as described in Table 1. The inflaction factor is estimated at $\psi = 10.8 \pm 8.2$, and Figure S14 illustrates that with large ψ, the behavior of spatially homogeneous model is similar to the behavior of the spatially heterogeneous SEIRS model. At smaller inflation factors, the spatially homogeneous model fails to reproduce the *divergence* of H3N2's HA phylogeny. The Dutch population size is too small to reproduce the basic features of the HA phylogeny, and the large inflation factor suggests that a viral reservoir with a population around 1.7×10^8 individuals is needed to match the basic features of H3N2 surveillance and molecular genetic data in Table 1. This is well in line with the estimated size of the source population under the spatial SEIRS model, $N^{\circlearrowleft} = 1.85 \pm 1.2 \times 10^8$.

Table S4 lists the estimated parameters and summary errors. In particular, the 95% credibility interval of the amplitude of seasonal forcing φ is [0.16, 0.6]. In the spatial SEIRS model, we found that the strength of seasonal forcing is difficult to estimate because it is correlated with the number of infected travelers from the source population, $m^{\downarrow} I^{\circlearrowleft}/N^{\circlearrowleft}$, which depends itself on $m^{\downarrow}$ as well as further epidemiological variables. Thus, we took the 95% credibility interval of φ to define a plausible parameter range for $\varphi^{\downarrow}$, see Table 2.

Table S4. Estimated model parameters and summary errors under the spatially homogeneous model (S4)**.**

	**prior density**	**mean±std. dev. and 95% conf. interval of estimated model parameters**		**mean±std. dev. and 95% conf. interval of estimated summary errors**
R_0	uninformative	2.58±0.45, [1.81, 3.25]	*μ-attack*	-0.68±0.43, [-1.24, 0.13]
R_{eff}	-⋆	1.12±0.29, [0.77, 1.69]	*σ-attack*	-0.37±0.27, [-0.69, 0.16]
$1/\phi$	0.9		*correlation*	-0.84±0.01, [-0.85,-0.83]
$1/\nu$	1.8		*explosiveness*	-0.22±0.14, [-0.54, 0.04]
$1/\gamma$	uninformative	8.3±1.8, [5, 10.9]	*pop-attack*	0.01±0.03, [-0.06, 0.05]
ρ	uninformative	0.16±0.07, [0.06,0.27]	*lineages*	-1.29±0.19, [-1.61, -0.99]
s	uninformative	0.14±0.2, [0.01,0.66]	*divergence*	0.22±0.12, [-0.02, 0.38]
ι_s	uninformative	1±0, [1,1]	*diversity*	-0.41±0.15, [-0.59, -0.12]
ζ	uninformative	2.7±1.0, [1.6, 4.2]	*TMRCA*	-0.95±0.18, [-1.26, -0.68]
N	fixed to Dutch demographic data, http://statline.cbs.nl			
μ	fixed to Dutch demographic data, http://statline.cbs.nl			
φ	uninformative	0.36±0.14, [0.16, 0.6]		
m	$\mathcal{U}(3 \times 10^6, 15 \times 10^6)$; encompassing lowest & highest annual records; http://statline.cbs.nl	10.9±3.2, [8.5, 15] $\times 10^6$		
ψ	$\mathcal{U}(1 \times 10^8, 30 \times 10^8)$; bounded above to keep simulations tractable	10.8±8.2, [1, 28] $\times 10^8$		

⋆ R_{eff} is not a model parameter and calculated from simulated incidence time series.

S.9 Sensitivity analyses

Our prior assumptions on the model parameter are listed in Table 2, along with a brief justification. Here, we describe how sensitive our results in the main text are to changes in the generation time $1/\phi + 1/\nu$, the birth rate $\mu^{\circlearrowleft}$, the functional form of the antigenic emergence rate h in Eqns. 5, and $\varphi^{\circlearrowleft}$.

Most importantly, we assume that the strength of seasonal forcing in the source population is weak, $\varphi^{\circlearrowleft} \in [0, 0.02]$. While plausible, there is considerable uncertainty in the strength, timing and form of influenza's seasonality in tropical regions [18]. Here, we show that the epochal evolution model of major antigenic clusters is well in agreement with the summaries in Table 1 if strong seasonal forcing is assumed in the source population, $\varphi^{\circlearrowleft} > 0.15$. Essentially, strong $\varphi^{\circlearrowleft}$ leads to sufficiently severe genetic bottlenecks in which less favorable

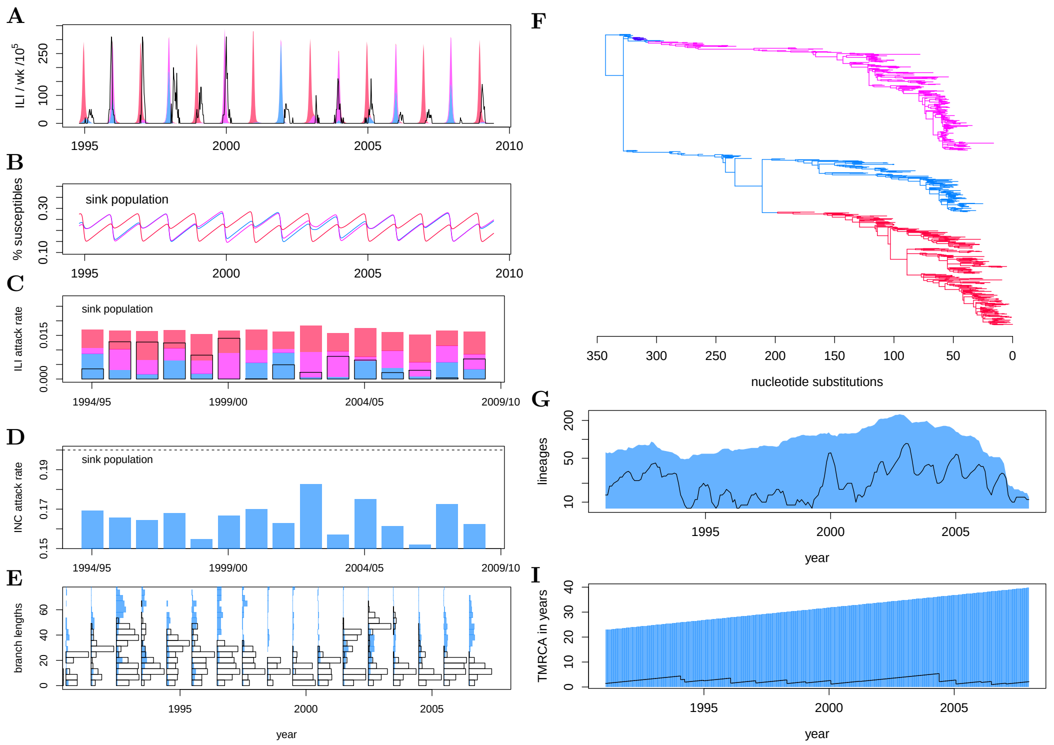

Figure S15. Phylodynamics under the spatial epochal evolution model with weak seasonal forcing in the source population, $\varphi^{\circlearrowright} = 0.03$**.** (A) Reported H3N2 incidence time series in the sink population, by week. Observed data is shown in black and simulated data in colors, with each color representing a major antigenic cluster. (B) Corresponding weekly time series of the percentage susceptibles in the sink population. (C) Simulated and observed case report seasonal attack rates, and (D) population level incidence attack rates in the sink population. Simulated data now shown in blue. (E) Histogram of pairwise nucleotide substitutions among sequences collected in the same season. (F) Simulated HA phylogeny. (G) Simulated and observed monthly time series of the number of lineages, and (I) simulated and observed monthly time series of $TMRCA$'s of all phylogenetic lineages circulating in the same month.

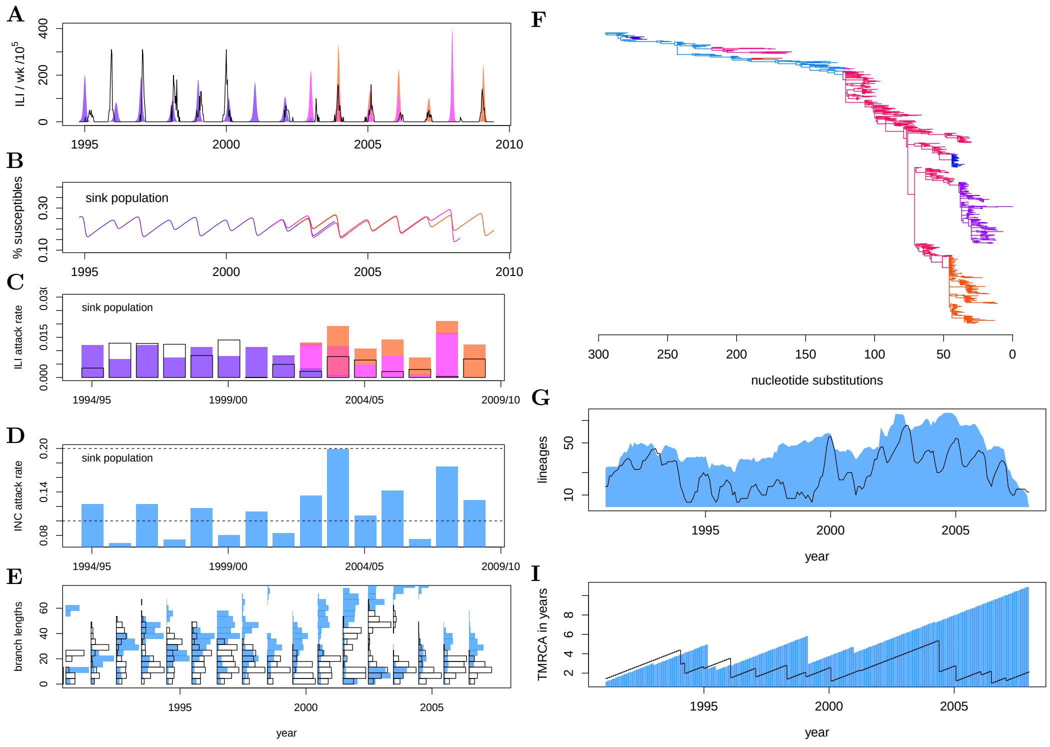

Figure S16. Phylodynamics under the spatial epochal evolution model with weak seasonal forcing in the source population, $\varphi^{\circlearrowright} = 0.05$. (A-I) as in Figure S15.

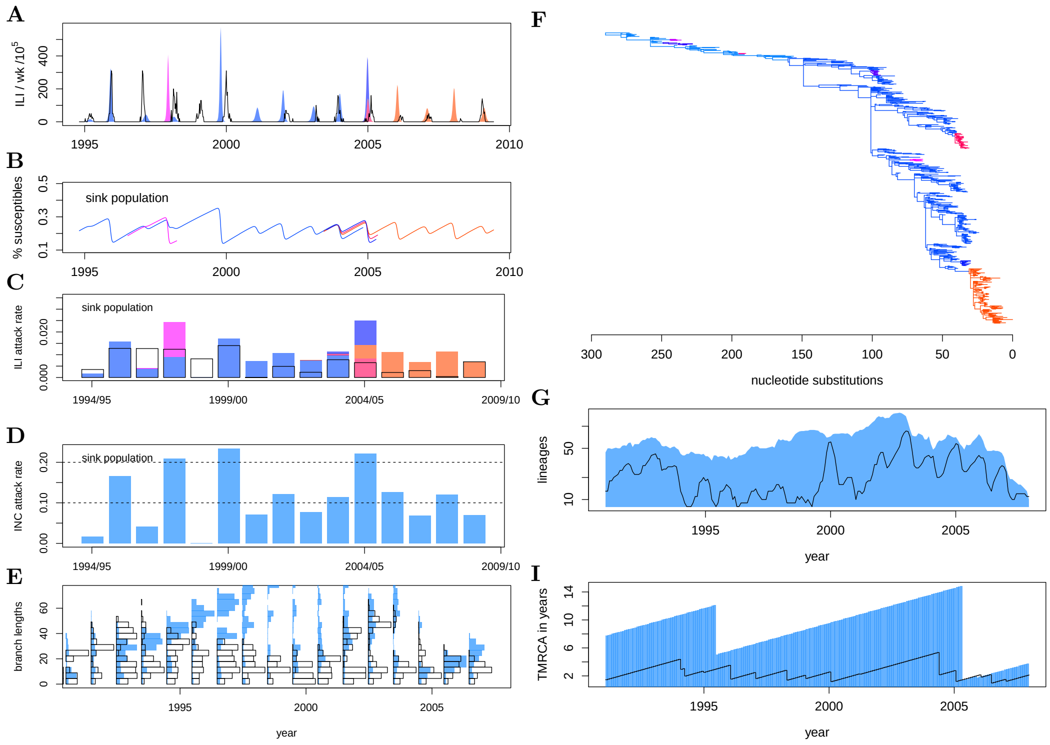

Figure S17. Phylodynamics under the spatial epochal evolution model with weak seasonal forcing in the source population, $\varphi^{\circlearrowleft} = 0.07$. (A-I) as in Figure S15.

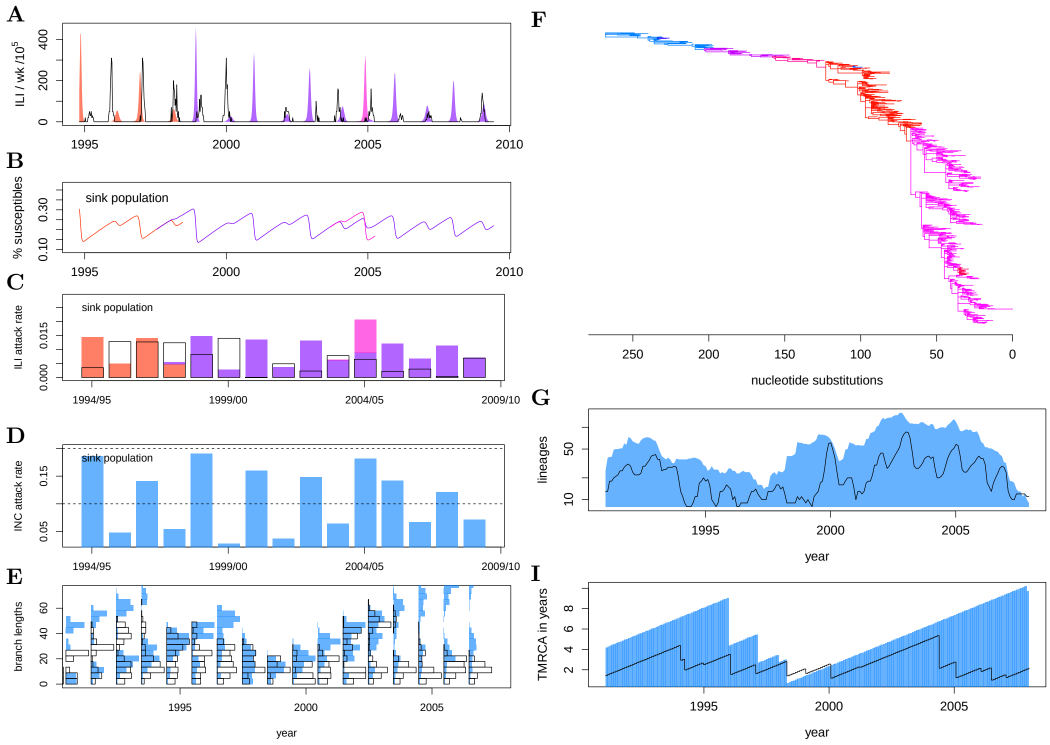

Figure S18. Phylodynamics under the spatial epochal evolution model with weak seasonal forcing in the source population, $\varphi^{\circlearrowleft} = 0.1$. (A-I) as in Figure S15.

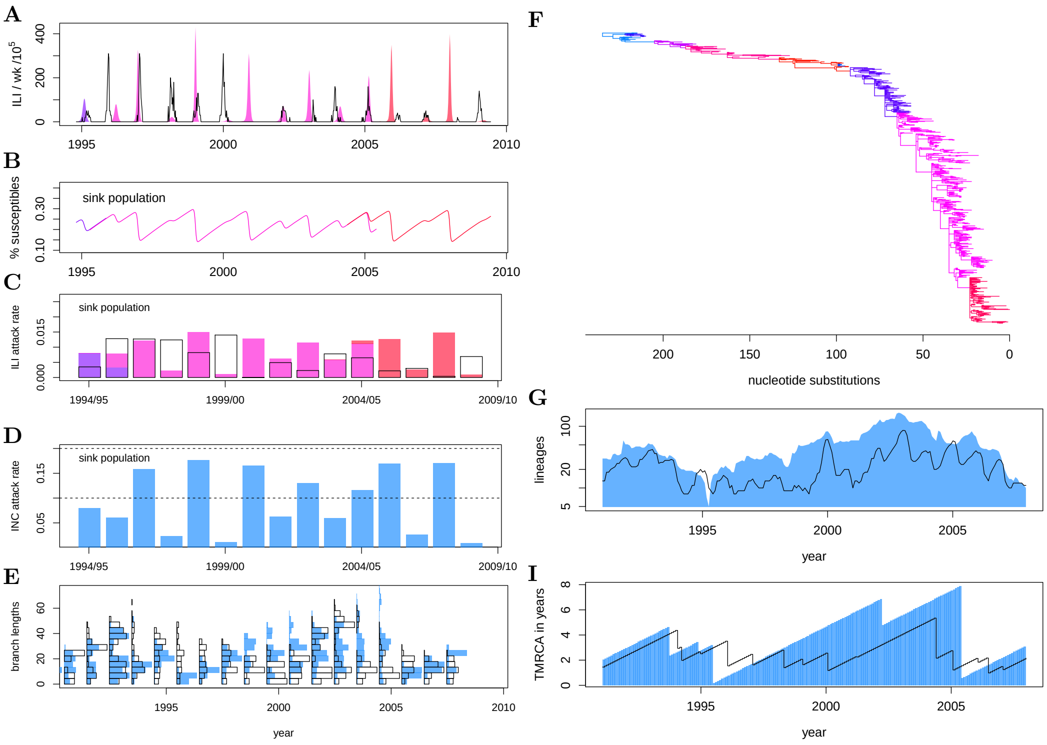

Figure S19. Phylodynamics under the spatial epochal evolution model with weak seasonal forcing in the source population, $\varphi^{\circlearrowleft} = 0.15$. (A-I) as in Figure S15.

Table S5. Sensitivity of parameter estimates to alternative generation times and different forms of the antigenic emergence rate function.

**model**	SEIRS	SEIRS	SEIRS	epochal	epochal	epochal
**fixed**						
$1/\phi$	1.8	2.3	2.7	1.8	2.3	2.7
$1/\nu$	0.9	1.2	1.3	0.9	1.2	1.3
κ	-	-	-	1	1	1
**estimated**			**mean±std. dev. and 95% conf. interval**			
R_0	2.4±0.8	2.5±0.9	2.7±1	16.7±4.5	18.7±5.6	20.7±5
	[1.4, 3.7]	[1.1, 4.6]	[1.2, 4.6]	[7.4, 25]	[7, 29]	[10, 30]
$1/\gamma$	9.1±2.7	8.9±2.8	8.9±2.8	228±97	240±96	235±92
	[4.6, 14]	[4.3, 14.6]	[4, 14.7]	[51, 390]	[73, 390]	[71, 391]
$\sigma_{i-1,i}$	-	-	-	0.81±0.05	0.79±0.07	0.77±0.06
	-	-	-	[0.71, 0.89]	[0.66, 0.92]	[0.66, 0.86]
ρ	0.16±0.08	0.17±0.07	0.17±0.07	0.63±0.21	0.57±0.22	0.6±0.2
	[0.06, 0.31]	[0.05, 0.29]	[0.05, 0.32]	[0.26, 0.97]	[0.21, 0.95]	[0.24, 0.97]
**model**	epochal	epochal	epochal	epochal	epochal	epochal
**fixed**						
$1/\phi$	1.8	2.3	2.7	1.8	2.3	2.7
$1/\nu$	0.9	1.2	1.3	0.9	1.2	1.3
κ	2	2	2	2⋆	2⋆	2⋆
**estimated**			**mean±std. dev. and 95% conf. interval**			
R_0	16.6±4.3	18.7±5.2	21.1±4.6	17.8±4.5	18.6±5.8	20.2±5
	[7.5, 25]	[8.2, 28.5]	[11.6, 29]	[8.6, 26]	[7.3, 28]	[9.4, 29.3]
$1/\gamma$	220±103	230±102	244±92	219±104	205±95	215±104
	[60, 391]	[38, 387]	[70, 395]	[38, 388]	[55, 380]	[31, 386]
$\sigma_{i-1,i}$	0.78±0.05	0.77±0.06	0.74±0.06	0.73±0.05	0.71±0.05	0.70±0.05
	[0.68, 0.88]	[0.65, 0.86]	[0.63, 0.84]	[0.63, 0.8]	[0.62, 0.81]	[0.61, 0.83]
ρ	0.58±0.22	0.56±0.22	0.56±0.21	0.63±0.23	0.62±0.22	0.5±0.23
	[0.22, 0.98]	[0.2, 0.96]	[0.22, 0.97]	[0.2, 0.98]	[0.22, 0.97]	[0.16, 0.98]

⋆ In these cases, the antigenic emergence rate (S5) was used.

antigenic variants go readily to extinction. Therefore, and in contrast to the analysis in the main text, the duration of cluster-specific immunity $1/\gamma$ is not constrained by the shape of the phylogeny and can be set small. Figure S15-S19 illustrate phylodynamics under the epochal evolution model with the parameters $R_0 = 3$, $1/\gamma = 9$, $1/\phi = 0.9$, $1/\nu = 1.8$, $\rho = 0.1$, $N^{\circlearrowleft} = 2 \times 10^8$, $1/\mu^{\circlearrowleft} = 50$, $\varphi^{\downarrow} = 0.25$, $m^{\downarrow} = 9.7 \times 10^6$, $m^{\circlearrowleft} = 0.05$, $\zeta = 3.3$, $s = 0.09$, $\kappa = 2$, $\lambda = 180$ but varying $\varphi^{\circlearrowleft}$. For $\varphi^{\circlearrowleft} > 0.15$, $1/\gamma$ can be relatively short, so that R_0 can be much smaller too and hence the simulated *pop-attack* are within the range of empirical estimates.

Recent reanalyses of influenza infections in household studies estimate a generation time around 2.7 days, while higher estimates around 4 days have also been reported [14, 19]. To evaluate if higher generation times up to 4 days could influence the fit and the goodness of fit of the both models considered, we initially considered the first tier only. The first tiers

of both models were fitted to the epidemiological summaries in Table 1, using the same respective tolerances and weighting schemes. In order to fit the epochal evolution model without computationally expensive phylogenetic simulations, we also generated the annual time series of the number of coexisting antigenic clusters and computed their average value in 1968-2002. To favor strain replacement, on average no more than 1.5 clusters were allowed to coexist in any season. Table S5 demonstrates that higher mean generation times $1/\phi + 1/\nu = 2.7, 3.5, 4$ result in slightly larger estimates of R_0, and do not affect the fit to any of the other parameters that can be estimated with the epidemiological summaries. Goodness of fit was insensitive to changes in the generation time. Consequently, full phylodynamic inference including phylogenetic summaries was not run.

Recent models of influenza evolution and epidemiology assume that antigenic variants emerge at a rate h that is either constant or increases through time [20, 21]. Following the argument in [20], it is plausible that h might alternatively depend on cumulative incidence rather than time, i.e.

$$h(t, t_i^e) = \frac{\kappa}{\lambda} \left(\frac{\sum_{s=t_i^e}^{t} I_i^+(s)}{\lambda} \right)^{\kappa - 1}. \tag{S5}$$

To evaluate if a constant antigenic emergence rate or dependence of h on cumulative incidence might influence the fit and the discrepancies of the fitted epochal evolution model, we initially considered only the first tier for computational reasons. As above, we required that the average number of coexisting antigenic clusters stays below 1.5. Table S5 illustrates that the key epidemiological parameters that can be estimated with the epidemiological summaries in Table 1 are not sensitive to using $\kappa = 1$ or (S5).

Finally, we also analyzed if a higher birth rate in the source population changes parameter inference or model assessment. Briefly, a lower birth rate $\mu^{\circlearrowleft}$ increases the extinction probability of phylodynamic simulations, especially for the fitted epochal evolution model, where infrequent but relatively strong cluster invasions lead to pronounced genetic bottlenecks, see Figure 6H. For an average lifespan of 80 years, more than 50% of all model simulations go extinct. A lower average lifespan than the 50 years used here reduced the extinction probability of phylodynamic simulations, but implied *lineages* that were 2-3 times as thick as in the observed HA phylogeny.

S.10 Supplementary Figures

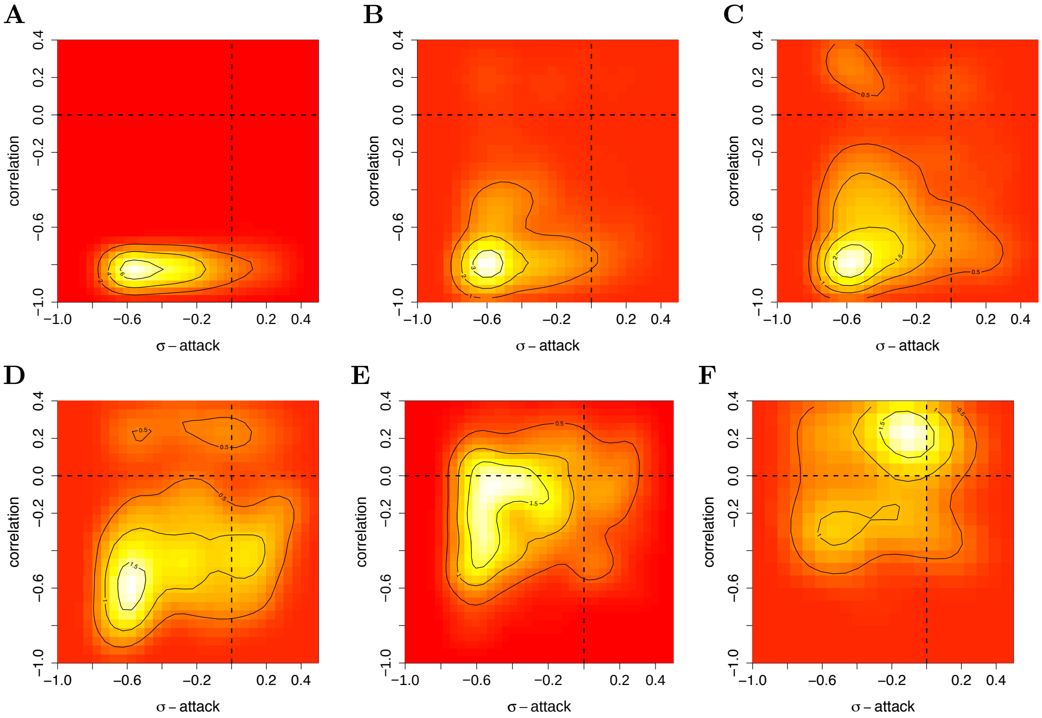

Figure S20. Reproducibility of interannual variability in H3N2 incidence for increasing ***nclust*** **in 1968-2002.** The first tier of the epochal evolution model was fitted with ABC to features of H3N2 surveillance data as specified in Table 1 under different assumptions on the number of antigenic clusters in 1968-2002: (A) $nclust = 0$, (B) $nclust = 3 \pm 1$, (C) $nclust = 5 \pm 1$, (D) $nclust = 7 \pm 1$, (E) $nclust = 11 \pm 2$, (F) $nclust = 17 \pm 4$. (A) is the same as Figure 3G, and (E) is the same as Figure 5I. To circumvent computationally expensive phylogenetic simulations, we here generated the annual time series of the number of coexisting antigenic clusters and computed their average value in 1968-2002. To favor strain replacement, on average no more than 1.5 clusters were allowed to coexist in any season. Four MCMC chains were run in parallel, burn-in periods were pruned and the remaining samples were pooled. The two-dimensional histograms between *correlation* and σ*-attack* indicate that the interannual variability in H3N2 incidence is increasingly better reproduced with a larger number of antigenic clusters that replace each other. Under the epochal evolution model, the turnover of more than 10 clusters, or equivalently 1 replacement event every 3-4 years, results in irregular incidence time series that are consistent with H3N2's interannual variability.

References

1. Dijkstra F, Donker G, Wilbrink B, Van Gageldonk-Lafeber A, Van Der Sande M, et al. (2009) Long time trends in influenza-like illness and associated determinants in The Netherlands. Epidemiol Infect 137: 473–9.

2. Meijer A, Rimmelzwaan G, Dijkstra F, Donker G (2009) Actuele ontwikkelingen betreffende influenza; griepspotters in actie. Tijdschr Infect 4: 176-84.

3. Thurston S, Wand M, Wiencke J (2000) Negative binomial additive models. Biometrics 56: 139–144.

4. Thompson W, Weintraub E, Dhankhar P, Cheng P, Brammer L, et al. (2009) Estimates of US influenza-associated deaths made using four different methods. Influenza and other respiratory viruses 3: 37–49.

5. Gilca R, De Serres G, Skowronski D, Boivin G, Buckeridge D (2009) The need for validation of statistical methods for estimating respiratory virus–attributable hospitalization. American Journal of Epidemiology 170: 925–936.

6. Donker G, Gravestein J (2007) De beste tijd voor griepvaccinatie. Huisarts & Wetenschap 50: 41.

7. Marin JM, Pudlo P, Robert C, Ryder R (2011) Approximate Bayesian computational methods. Statistics and Computing : 1-14.

8. Marjoram P, Molitor J, Plagnol V, Tavaré S (2003) Markov Chain Monte Carlo without likelihoods. Proc Natl Acad Sci USA 100: 15324-15328.

9. Toni T, Welch D, Strelkowa N, Ipsen A, Stumpf MPH (2008) Approximate Bayesian computation scheme for parameter inference and model selection in dynamical systems. Journal of The Royal Society Interface 6: 187-202.

10. Gilks WR, Richardson S, Spiegelhalter DJ (1996) Markov Chain Monte Carlo in practice. Chapman & Hall.

11. Brooks S, Roberts G (1997) Assessing convergence of Markov Chain Monte Carlo algorithms. Technical report, University of Bristol.

12. Sisson SA, Fan Y, Tanaka MM (2007) Sequential Monte Carlo without likelihoods. Proc Natl Acad Sci USA 104: 1760-1765.

13. Ratmann O, Pudlo P, Richardson S, Robert C (2011) Monte carlo algorithms for model assessment via conflicting summaries. Arxiv preprint arXiv:11065919 .

14. Ferguson N, Cummings D, Cauchemez S, Fraser C, Riley S, et al. (2005) Strategies for containing an emerging influenza pandemic in Southeast Asia. Nature 437: 209–214.

15. Wallinga J, Lipsitch M (2007) How generation intervals shape the relationship between growth rates and reproductive numbers. Proceedings of the Royal Society B: Biological Sciences 274: 599.

16. Cauchemez S, Ferguson N (2008) Likelihood-based estimation of continuous-time epidemic models from time-series data: application to measles transmission in London. Journal of the Royal Society Interface 5: 885–897.

17. Cox N, Subbarao K (2000) Global epidemiology of influenza: past and present. Annual Review of Medicine 51: 407–421.

18. Viboud C, Alonso W, Simonsen L (2006) Influenza in tropical regions. PLoS medicine 3: e89.

19. Monto A, Koopman J, Longini Jr I (1985) Tecumseh study of illness. xiii. influenza infection and disease, 1976–1981. American Journal of Epidemiology 121: 811–822.

20. Koelle K, Khatri P, Kamradt M, Kepler T (2010) A two-tiered model for simulating the ecological and evolutionary dynamics of rapidly evolving viruses, with an application to influenza. Journal of The Royal Society Interface 7: 1257–1274.

21. Bedford T, Rambaut A, Pascual M (2012) Canalization of the evolutionary trajectory of the human influenza virus. BMC Biology 10.
